# Temperature and genetic background drive mobilization of diverse transposable elements in a critical human fungal pathogen

**DOI:** 10.1101/2025.05.19.654958

**Published:** 2025-05-23

**Authors:** Anna I. Mackey, Vesper Fraunfelter, Samantha Shaltz, John McCormick, Callan Schroeder, John R. Perfect, Cedric Feschotte, Paul M. Magwene, Asiya Gusa

**Affiliations:** a Department of Molecular Genetics and Microbiology, Duke University, Durham, NC, 27701, USA.; b Department of Molecular Biology and Genetics, Cornell University, Ithaca, NY, 14850.; c Department of Medicine, Duke University, Durham, NC, 27710, USA.; d Department of Biology, Duke University, Durham, NC, 27710, USA.

## Abstract

Transposable elements (TEs) are key agents of genome evolution across all domains of life. These mobile genetic elements can cause mutations through transposition or by promoting structural rearrangements. Stress conditions can amplify TE activity, either by impairing TE suppression mechanisms or through stress-induced interactions between transcription factors and TE sequences, offering a route for rapid genetic change. As such, TEs represent an important source of adaptability within populations. To investigate the interplay between environmental stress and eukaryotic TE dynamics relevant to infectious disease, we examined how heat stress and nutrient limitation affect TE mobility in the human fungal pathogen *Cryptococcus neoformans*, using a collection of clinical and environmental isolates. Seven distinct mobile element families, encompassing diverse retrotransposons and DNA transposons, were captured mobilizing to confer antifungal resistance, including a novel element, CNEST, which belongs to the CACTA, Mirage, Chapaev (CMC) supergroup. Heat stress at human body temperature (37°C) significantly increased the mobilization of a subset of these TEs, leading to higher rates of acquired antifungal resistance. Whole-genome assemblies revealed that, compared to retrotransposons, DNA transposons were hypomethylated and approximately uniformly distributed throughout the genome, features that may contribute to their frequent mobilization. We further assessed TE-driven genomic changes within hosts using serial isolates from patients with recurrent cryptococcal infections and from isolates passaged through mice. While we observed evidence of TE copy number changes near chromosome ends, we found no indication of TE-mediated alterations near gene-coding regions across any of the serial isolates. Finally, TE mobility was isolate- and strain-dependent, with significant variation even among clonally related isolates collected from the same patient, emphasizing the role of genetic background in shaping TE activity. Together, these findings reveal a complex, dynamic relationship between environmental stress, genetic background, and TE mobility, with important implications for adaptation and acquired antifungal resistance in *C. neoformans*.

## INTRODUCTION

Transposable elements (TEs) are powerful drivers of genomic change and provide a mechanism for adaptive evolution in fungal pathogens (1–8). TEs are mobile genetic elements that fall into two broad categories: class I retrotransposons, which mobilize via an RNA intermediate through a “copy-and-paste” mechanism, and class II DNA transposons, which mobilize via a DNA intermediate, typically through a “cut-and-paste” mechanism (9). TE activity can lead to genomic instability through unchecked transposition or by providing homologous sequences throughout the genome that promote rearrangements (10). However, TEs can also promote beneficial genetic changes, especially under changing or stressful environmental conditions, by modulating the expression of nearby genes, disrupting endogenous gene functions, or providing beneficial cargo (10–14). TE-mediated genetic changes in fungi have been linked to various adaptive phenotypes, including increased fitness under heavy metal stress in *Schizosaccharomyces pombe* and nitrogen-limited conditions in *Saccharomyces cerevisiae* (1–5). Additionally, various stressors can increase TE expression and mobilization rates (14–17).

Diverse TEs are present in *Cryptococcus neoformans*, a human fungal pathogen which primarily causes disease in immunocompromised individuals such as cancer patients, organ transplant recipients, and people living with HIV/AIDS (18–20). *C. neoformans* is responsible for approximately 147,000 deaths annually and accounts for up to 20% of AIDS-related mortality (21,22). It is a ubiquitous environmental yeast, and inhalation of spores or desiccated yeast cells can lead to pulmonary infection that may disseminate across the blood-brain barrier, causing cryptococcal meningitis (18). Approximately 10 – 20% of patients diagnosed with cryptococcal meningitis experience recurrent disease, which can persist for years despite antifungal treatment (23). In-host microevolution can result in adaptive genetic changes that enhance antifungal resistance, alter virulence traits, or increase overall pathogenicity, contributing to persistent infections and treatment failure (24–27). Although in-host microevolution influences disease outcomes, the genetic basis of altered virulence traits and antifungal resistance often remains unknown, potentially because short-read sequencing and variant calling, the primary detection methods, are less suited for identifying structural rearrangements and TE polymorphisms (28).

During infection, *C. neoformans* encounters a variety of stressors during the environment-to-host transition. These include shifts in nutrient availability, pH, exposure to reactive oxygen species (ROS), and elevated temperature (37°C) (29). Previous studies in the less pathogenic sister species *Cryptococcus deneoformans* have shown that heat stress significantly increases TE mobilization rates, exceeding those of single nucleotide polymorphisms (SNPs) and insertions/deletions (indels) by more than threefold per generation during in vitro passaging at 37°C (30,31). These findings suggest that in stressful environments, TEs can be the primary drivers of genomic change. Indeed, TE mobilization in *C. deneoformans* has been observed during murine host infection and, in both *C. neoformans* and *C. deneoformans*, has been linked to the development of resistance to 5-fluorocytosine (5-FC), a first-line antifungal treatment (30–32). However, it is not known whether heat stress increases TE mobilization rates in *C. neoformans*, which is responsible for approximately 95% of cryptococcal infections (33), and whether TEs contribute to genetic changes accumulated during persistent human infection.

*C. neoformans* is known to harbor a variety of TEs, including DNA transposons as well as long terminal repeat (LTR) and non-LTR retrotransposons (19,20). The best-characterized TE suppression mechanism in *C. neoformans* is RNA interference (RNAi). Through this pathway, transcripts targeted for silencing are processed into small interfering RNAs (siRNAs), which are loaded into the RNA-induced silencing complex (RISC) to target homologous transcripts for degradation (34–37). RNAi has been shown to silence double-stranded RNAs, transcripts with sub-optimal splicing signals, repetitive transgenes, and TEs in *C. neoformans* (34,35,37,38). Unlike in the yeast *S. pombe*, RNAi is strictly involved in post-transcriptional silencing in *C. neoformans* and does not lead to transcriptional silencing by facilitating the assembly or maintenance of heterochromatin (34). Prior studies have shown that loss-of-function mutations in key RNAi genes can lead to a hypermutator phenotype, driven by rampant TE mobilization, but only in strains with high TE burden (32,38). TE mobilization may also be shaped by epigenetic context. LTR and non-LTR elements are enriched in centromeric and subtelomeric regions (38,39), where they co-localize with epigenetic features of silent chromatin, such as 5-methylcytosine DNA methylation and repressive histone modifications, including H3K27me3 and H3K9me2 (38–42). These marks are also enriched in centromeric and subtelomeric regions, and are rarely detected in gene-rich chromosomes arms (38–42). Despite this association, the regulatory effects of DNA methylation and heterochromatin on TEs in *C. neoformans* remain poorly understood, particularly regarding their influence on the mobilization of active TE families.

In this study, we investigated how stressors encountered during human infection, including elevated temperature and limited nutrient availability, influence TE mobilization in *C. neoformans*. Using a reporter gene-based approach, we examined TE mobility in genetically diverse clinical and environmental isolates, including representatives from three of the four major lineages (VNI, VNII, and VNBII) (43). We found diverse, active TEs in *C. neoformans*, including several whose identity or mobility were previously unknown. TE mobility was temperature-dependent for all elements assessed, with heat stress significantly increasing transposition rates for a subset of elements. De novo telomere-to-telomere genome assemblies were used to examine TE-driven genetic changes in serially collected isolates from patients with recurrent cryptococcal meningitis. Using these assemblies, we annotated TEs to assess the genomic distribution and DNA methylation status of various transposable element types. DNA transposons were approximately uniformly distributed throughout the genome and had significantly reduced levels of DNA methylation compared to retrotransposons. Lastly, we found that TE copy number and mobility were highly isolate- and strain-dependent, suggesting that genetic background is a major determinant of TE-mediated genomic impacts. Together, these results highlight the critical roles of environmental factors, particularly temperature, in driving stress-induced genomic changes and adaptive evolution via transposition in the human fungal pathogen *C. neoformans*.

## RESULTS

### Diverse transposable elements mobilize in a subset of *C. neoformans* clinical isolates

**Figure 1.**
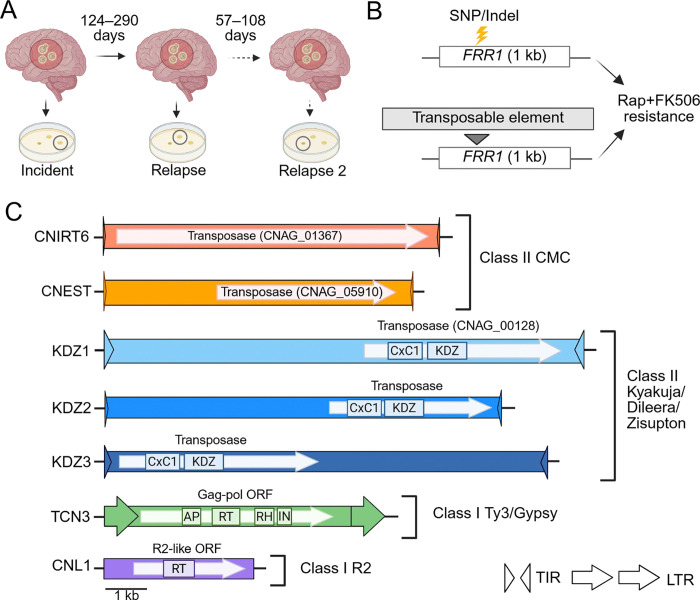
Diverse transposable elements (TEs) captured mobilizing in clinical isolates of *C. neoformans*. (**A**) Schematic of clinical isolate collection from patients with recurrent cryptococcal meningitis at disease onset and after subsequent relapse(s). Single colonies were isolated from patient cerebrospinal fluid. (**B**) Diagram of the “transposon trap” assay. Point mutations or TE insertions in the *FRR1* reporter gene confer resistance to rapamycin and FK506 (rap+FK506). (**C**) Illustration of seven mobile TEs, including class II elements from the CMC (CACTA, Mirage, Chapaev) supergroup and Kyakuja/Dileera/Zisupton superfamily, and class I elements from the Ty3/gypsy and R2 superfamilies. Terminal inverted repeats (TIRs) and long terminal repeats (LTRs) are indicated by triangles and arrows, respectively. Genes within TEs that are predicted to facilitate mobilization are shown as white arrows. Functional domains are indicated, including KDZ (Kyakuja/Dileera/Zisupton transposase), CxC1 (predicted zinc chelating domain), AP (aspartic protease), RT (reverse transcriptase), RH (RNase H), and IN (integrase). TEs are drawn to scale.

To investigate transposable element (TE) mobilization dynamics in *Cryptococcus*, we obtained a set of 33 clinical isolates from 18 South African patients with recurrent cryptococcal meningitis, collected as part of the GERMS-SA surveillance study (Table S1) (44). Isolates were collected by plating cerebrospinal fluid and selecting a single colony at the time of diagnosis (incident) and after subsequent disease relapse(s), which occurred 57 to 238 days later (Fig 1A, Table S1). Using short read sequencing, a prior study found that isolates collected from the same patient differed by few single nucleotide polymorphisms (SNPs) and insertions/deletions (indels), and that these isolates formed monophyletic clades in phylogenetic analyses, suggesting that disease relapses were clonal and not due to reinfection (24). Isolates are genetically diverse, including *Cryptococcus neoformans* isolates belonging to three of the four major clades, VNI, VNII, and VNBII, and *Cryptococcus gattii* isolates belonging to clades VGI and VGIV (Table S1) (43,45).

Prior work from our group showed that growth at 37°C increased TE mobilization rates in the less pathogenic species *Cryptococcus deneoformans* (30,31). To investigate TE activity under heat stress in *C. neoformans* and *C. gattii*, both recognized as priority fungal pathogens by the World Health Organization (46), we conducted a preliminary screen using a “transposon trap” assay that detects mutations in the *FRR1* reporter gene, a system previously used by our group and others (30,32,38). Incident and relapse isolates from the 18 South African cases were cultured in non-selective nutrient-rich YPAD medium at 37°C and plated to medium containing the antifungal drugs rapamycin and FK506 (rap+FK506). Only mutations in *FRR1*, which encodes the shared drug target, can confer resistance to both antifungals (Fig 1B) (47). Ten independent rap+FK506 resistant mutants were collected from each isolate and analyzed by PCR and sequencing to determine the causative mutation of drug resistance. Large DNA insertions, approximately 150 bp – 11 kb, detected by PCR amplification and indicative of TE insertions were confirmed by Sanger sequencing. Isolates from three cases contained TE insertions disrupting the *FRR1* locus at 37°C: cases 7, 15, and 77, which belong to the VNBII, VNI, and VNII clades, respectively (Table 1). TE mobilization was detected exclusively in *C. neoformans*; of the three *C. gattii* cases screened, only point mutations were identified in *FRR1*. Subsequent screening of case 7 and 77 isolates at 30°C (see below) detected additional mobile element families.

In total, we screened over 1,000 independent rap+FK506-resistant mutants and identified more than 350 independent TE insertion events in *FRR1*. Elements were assigned to known families based on sequence similarity, using Sanger or linear amplicon sequences compared to *C. neoformans* TE family consensus sequences in Repbase, or to GenBank submissions when family-level consensus sequences were unavailable. Family membership was assigned based on the 80–80-80 rule: nucleotide similarity had to exceed 80% over at least 80 base pairs and cover at least 80% of the recovered sequence length (48). An exception to this was the non-LTR retrotransposon CNL1, which shares only ~60% nucleotide similarity with the CNL1 consensus sequence in Repbase. However, it exhibits high (> 99%) nucleotide similarity to an active mobile element previously classified as CNL1 in *C. neoformans* by Priest et al. (2022), so we have retained this name. Additionally, junction PCR was used to identify TE families for a subset of insertion events, employing primers designed to match the internal region of elements previously identified as inserting into *FRR1* in the same isolate. In total, we identified seven diverse TE families mobilizing in *C. neoformans* clinical isolates (Fig 1C). This includes an element belonging to a family that, to our knowledge, has not been identified in previous studies (CNEST), as well as elements not previously reported to be mobile (CNIRT6, TCN3, KDZ3) (Fig 1C, Table S2). These elements included DNA transposons (class II), as well as long terminal repeat (LTR) and non-LTR retrotransposons (class I).

### CNIRT6 transposition drives increased drug resistance rates at 37°C in case 15

**Figure 2.**
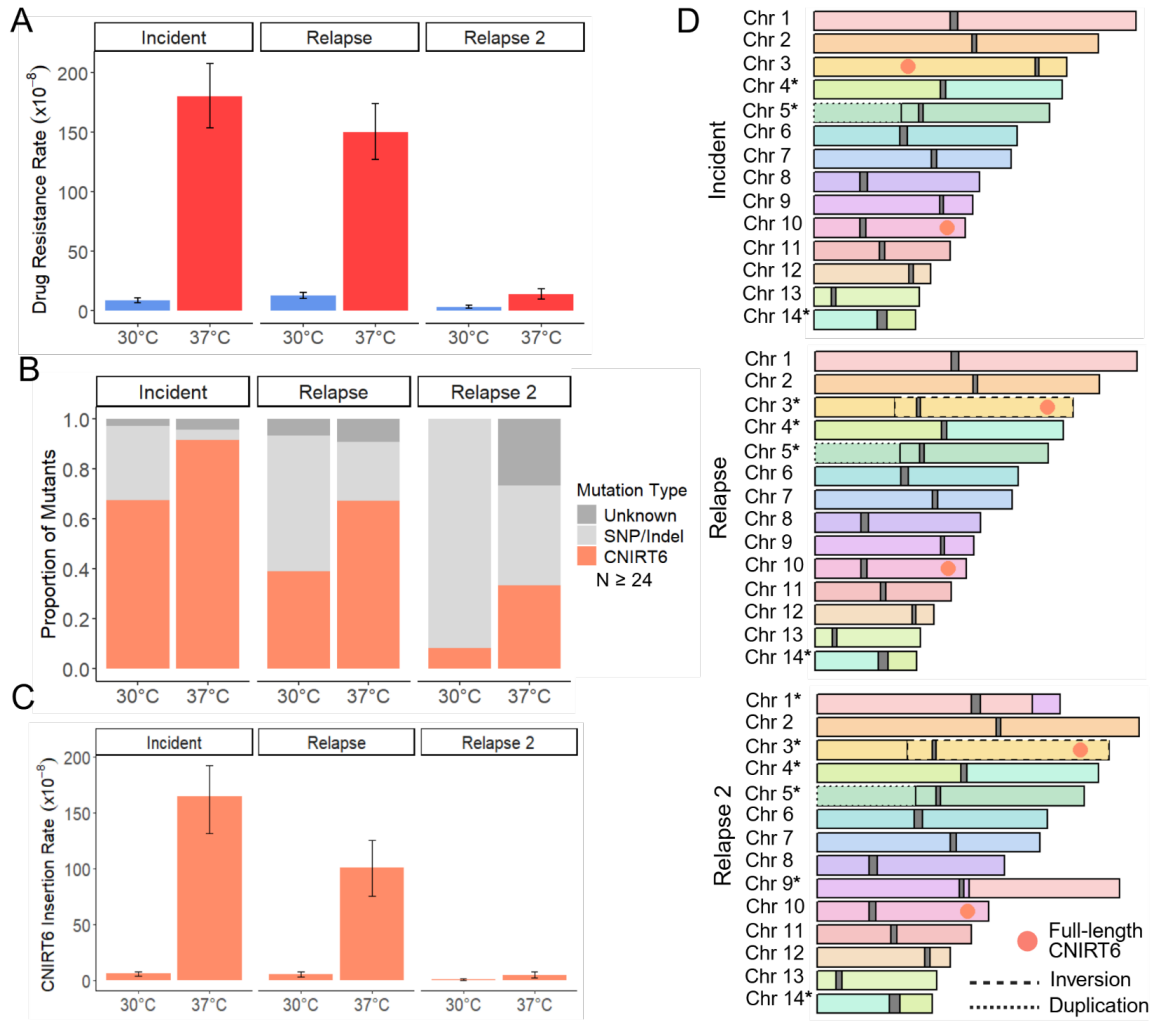
Mobilization of the DNA transposon CNIRT6 is induced by heat stress. (**A**) Rap+FK506 drug resistance rates following growth in non-selective YPD medium at 30°C or 37°C. (**B**) Mutation spectra in the *FRR1* gene for independent rap+FK506-resistant mutants. The mutation type “Unknown” indicates failure to amplify *FRR1* or absence of detectable mutations in its sequence (N = 24 – 55). (**C**) Insertion rate of CNIRT6 into *FRR1*. (**D**) Karyograms of the case 15 incident, relapse, and relapse 2 isolates. Full-length copies of CNIRT6 are shown as orange circles. Chromosome synteny is indicated by chromosome color, with inversions and duplications outlined in dashed and dotted lines, respectively. All chromosomes with identified structural rearrangements are marked with an asterisk. Error bars represent 95% confidence intervals (CIs); rates are considered statistically different if error bars do not overlap.

To assess how heat stress impacts rap+FK506 drug resistance rates, fluctuation assays were performed on case 15 incident, relapse, and relapse 2 isolates. All displayed significant increases in rap+FK506 resistance rates during growth at 37°C compared to 30°C in YPAD medium (21-, 12-, and 4-fold, respectively) (Fig 2A). The spectrum of mutations in *FRR1* was obtained by PCR and Sanger or linear amplicon sequencing of independent mutants, followed by mapping to the wild-type *FRR1* sequence for each isolate (Fig S1A). For a subset of insertions, junction PCR was also used. Strikingly, many of the mutations in *FRR1*, up to 91% in the incident isolate at 37°C, were caused by insertions of a large DNA transposon with 99.7% nucleotide similarity to a putative element named CNIRT6 (GenBank: JQ277267.1) (Fig 2B). CNIRT6 was originally identified in *C. neoformans* strain JF289 through sequence analysis, however, this transposon has not been described in the literature and is absent from Repbase. Mapping of a subset of these insertions revealed CNIRT6 transposition into *FRR1* introns and exons, resulting in 3 bp terminal site duplications (TSDs) with variable sequences (Fig S2A, B). CNIRT6 insertion rates were significantly higher at 37°C compared to 30°C, increasing by 28-, 20-, and 16-fold in the incident, relapse, and relapse 2 isolates, respectively (Fig 2C). Notably, drug-resistance rates at 37°C and CNIRT6 mobility at both temperatures decreased progressively in relapse isolates collected 166 and 223 days after the incident isolate.

Given the significant differences in CNIRT6 mobilization rates among case 15 isolates, we hypothesized that inter-isolate variability may be driven by differences in copy number, genomic position, or epigenetic regulation. To investigate these factors, and to assess all inter-isolate polymorphisms caused by mobile elements, we performed long-read sequencing to generate contiguous, telomere-to-telomere genome assemblies, which were polished with Illumina reads (Table S3). 5-methylcytosine (5mC) DNA methylation was identified using Nanopore signal data. Given that all CNIRT6 insertions into *FRR1* appeared to be the same length by gel electrophoresis and shared high nucleotide similarity to one another (> 99%), the full-length CNIRT6 sequence, obtained by linear amplicon sequencing from an insertion into *FRR1*, was used as the query in BLAST-like alignment tool (BLAT) (49) searches against case 15 genome assemblies to identify full-length copies (Fig S1A). For all BLAT searches predetermined cutoffs of nucleotide identity ≥ 90% and coverage ≥ 95% were used to call full-length copies. This analysis revealed two full-length CNIRT6 copies that are positionally conserved between incident and relapse isolates: one is an exact match to the CNIRT6 sequence identified inserting into *FRR1*, and the second differs by a single nucleotide substitution (Fig 2D). The nucleotide sequences of both CNIRT6 copies are identical between the serial isolates. No DNA methylation was detected in any full-length CNIRT6 copies across all case 15 isolates, as indicated by methylation frequencies < 0.75 at each CpG site, meaning fewer than 75% of long-read signal data supported a methylated call (Table S4). Therefore, differences in genomic copy number, chromosomal location, or DNA methylation do not appear to underlie differences in CNIRT6 mobilization rates between serial isolates.

To comprehensively assess all genomic changes between case 15 isolates that may have been mediated by TEs other than CNIRT6, we performed synteny and read depth analyses, and used Synteny and Rearrangement Identifier (SyRI) (50), to define structural rearrangements and identify insertions, deletions, and duplications. Repeat masking of insertions, deletions, and duplications with Repbase CENSOR revealed no evidence of transposition events between serial isolates. Relative to the genome reference strain H99, all three case 15 isolates contain a segmental duplication of ~614 kb of the left arm of chromosome 5 and a translocation between chromosomes 4 and 14 where the breakpoint lies in the predicted centromeres (Fig 2D). Rearrangements that occurred between serial isolates include an inversion of ~1.26 Mb on chromosome 3 in the relapse and relapse 2 isolates and a translocation between chromosomes 1 and 9 in the relapse 2 isolate (Fig 2D). Breakpoints for both inter-isolate rearrangements were outside of the predicted centromeres. No TE annotations were observed near rearrangement breakpoints, suggesting that another source of sequence homology or homology-independent DNA repair mechanisms mediated these rearrangements.

To further characterize the active DNA transposon CNIRT6, we performed sequence analysis and found that this element was ~7.5 kb in length and contained 24 bp terminal inverted repeats beginning with ‘CACTG’ (Fig 1C, Table S2). Though no CNIRT6 movement has been reported in the H99 reference strain, a BLAT search revealed two approximately full-length copies in H99 (identity ≥ 90% and coverage ≥ 95%), containing a ~6.9 kb gene annotation (CNAG_01367). Repeat masking of the predicted protein sequence with Repbase CENSOR revealed hits to transposases from TEs in the CACTA and EnSpm superfamilies, which contain a ‘CACTA’ motif at their distal ends, resembling the ‘CACTG’ motif found in CNIRT6 (51). To confirm that CNAG_01367 encodes a transposase and refine this classification, a multiple sequence alignment was performed using the amino acid sequences from CNAG_01367 and transposases from several “cut-and-paste” DNA transposons from the CMC (CACTA, Mirage, Chapaev) supergroup (52). This alignment revealed a conserved DDE motif, which catalyzes the “cut-and-paste” mechanism of transposition, indicating that CNIRT6 represents a full-length element predicted to encode a transposase of the CMC superfamily (Fig S3A). To examine the relationship of CNIRT6 to other CMC transposons, an unrooted tree was constructed using transposase amino acid sequences representative of CMC/EnSpm families from animals, plants, and fungi. CNIRT6 formed a statistically-supported clade (Bootstrap value 79%) with CACTA (also known as EnSpm) elements previously identified in other fungi, as well as plants and animals (Fig S3B). We conclude that CNIRT6 is a member of the CACTA superfamily within the CMC supergroup (52).

Lastly, given the robust activity of CNIRT6 in case 15 isolates grown in YPAD, we hypothesized that CNIRT6 may have mobilized during recurrent infections associated with other cases. To test this, we performed Southern blots on a subset of cases to identify putative transposition events between incident and relapse isolates. We generated a 551 bp CNIRT6-specific probe and utilized a restriction enzyme that cuts once within CNIRT6, outside of the probe site, and produces a range of fragment lengths separable by gel electrophoresis (Fig S4A). Consistent with whole-genome assembly comparisons, there was no difference in the hybridization band pattern for CNIRT6 among case 15 isolates (Fig S4B). However, several other patient cases displayed differences in CNIRT6 hybridization band patterns among serial isolates (Fig S4B). Case 1 was of particular interest, as it exhibited changes in the molecular weight of three bands, suggesting that these copies may occupy different genomic locations and indicating putative CNIRT6 transposition events between the incident and relapse isolates.

To investigate putative CNIRT6 transposition events in case 1 isolates, we generated whole-genome assemblies of the incident and relapse isolates and performed BLAT searches using full-length CNIRT6 as the query sequence. No full-length copies of CNIRT6 were detected in either genome; an identical number of truncated copies were present at the same genomic locations in both case 1 isolates (Fig S4C). Additionally, repeat masking of insertions, deletions, and duplications identified by SyRI revealed no evidence of TE mobilization events between the isolates. We conclude that the observed differences in Southern hybridization band patterns likely result from other genomic polymorphisms between the isolates, such as other genomic rearrangements or mutations introducing restriction site polymorphisms, highlighting a limitation of this approach in detecting transposition events. In the case 1 relapse isolate, for example, a translocation was detected between chromosomes 3 and 10, with the breakpoint located outside of the predicted centromere regions. There were no TEs near the breakpoint, suggesting that this rearrangement was not mediated by TE-derived sequence homology.

### CNEST transposition drives increased drug resistance rates at 37°C in case 77

**Figure 3.**
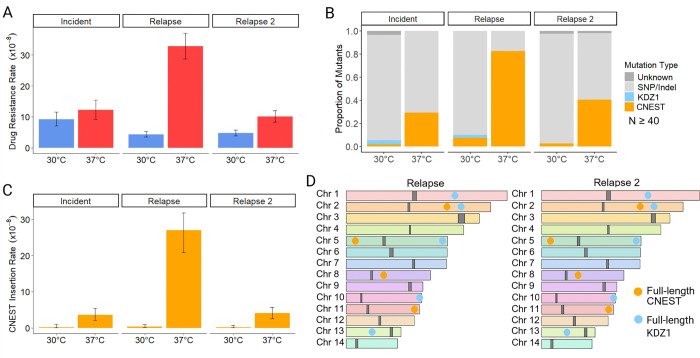
Mobilization of the DNA transposon CNEST is induced by heat stress. (**A**) Rap+FK506 drug resistance rates following growth in non-selective YPD medium at 30°C or 37°C. (**B**) Mutation spectra in the *FRR1* gene for independent rap+FK506-resistant mutants. The mutation type “Unknown” indicates failure to amplify *FRR1* or absence of detectable mutations in its sequence (N = 40 – 65). (**C**) Insertion rate of CNEST into *FRR1*. (**D**) Karyograms of the case 77 relapse and relapse 2 isolates. Full-length copies of CNEST (yellow) and KDZ1 (light blue) are shown as circles. Chromosome synteny is indicated by chromosome color. Error bars represent 95% confidence intervals (CIs); rates are considered statistically different if error bars do not overlap.

For case 77, the relapse, and relapse 2 isolates displayed significant increases in rap+FK506 drug resistance rates during growth at 37°C vs 30°C (Fig 3A). Of mutations in *FRR1* observed after growth at 37°C, 29 – 82% were caused by insertion of a novel DNA transposon, which we named CNEST (*C**ryptococcus* *n**eoformans* EnSpm-like Transposon; naming rationale described below) (Fig 3B). CNEST insertions appeared identical in length by gel electrophoresis and mapped to introns and exons in *FRR1* (Fig S1B, S5A). Insertions resulted in 2 bp ‘TA’ TSDs (Fig S5B). CNEST insertion rates were significantly higher for all isolates at 37°C; 22-, 83-, and 36-fold in the incident, relapse, and relapse 2, respectively (Fig 3C). The full-length CNEST sequence obtained by linear amplicon sequencing was ~7.2 kb in length, with 33 bp TIRs beginning with ‘CACGG’ (Fig 1C, Table S2).

Interestingly, we also identified insertions of the DNA transposon KDZ1, recently characterized by Huang et al. 2024, which occurred at a lower frequency and only during growth at 30°C (Fig 3B) (32). KDZ1 is an ~11 kb element with 241 bp TIRs, and its insertion into *FRR1* resulted in 8 bp TSDs (Fig S5B, Table S2). The KDZ1 putative transposase contains a catalytic core affiliated with the Kyakuja-Dileera-Zisupton (KDZ) superfamily and a predicted Zn-chelating CxC1 domain, both of which have been previously described in fungi (Fig 1C) (53).

Assemblies of the relapse and relapse 2 isolates revealed conserved copy number and position of CNEST and KDZ1 by BLAT analysis (identity ≥ 90% and coverage ≥ 95%) with 4 full-length copies of CNEST and 5 full-length copies of KDZ1 (Fig 3D). No DNA methylation was detected in any CNEST copies, as indicated by methylation frequencies < 0.75 at each CpG site from long-read signal data (Table S4). For KDZ1, a single copy contained one methylated CpG in the relapse (Table S4). In the relapse 2, two KDZ1 copies contained one methylated CpG. The additional methylated CpG in the relapse 2 isolate is ~900 bp upstream of the predicted transposase start codon; however, a single methylated CpG seems unlikely to impact transcription of the transposase.

No major genomic rearrangements were identified between the relapse and relapse 2 assemblies. Additionally, no other TE-mediated genomic changes were detected, as all insertions, deletions, and duplications identified by SyRI were repeat masked and showed no significant sequence similarity to TEs in the Repbase library. Although mutation rate and spectra data are provided for the case 77 incident isolate, there was some variability in the growth of independent colonies and some inconsistency in mutation rate observed between independent fluctuation assays. The likelihood of genome instability in this isolate led to its exclusion from nanopore sequencing and genome assembly.

To further characterize CNEST, which has not previously been identified as mobilizing in the H99 reference strain, we performed a BLAT homology search, which revealed two approximately full-length copies in H99 (≥ 90% identity and ≥ 95% coverage), containing a ~4.4 kb gene annotation (CNAG_05910). Repeat masking of CNEST and the predicted amino acid sequence of CNAG_05910 revealed no hits to TEs or transposases in the Repbase library. However, given the identification of CNIRT6 as a member of the CMC supergroup, we predicted that CNEST may also encode a CMC transposase. Multiple sequence alignment of the CNAG_05910 amino acid sequence with transposases of the CMC supergroup revealed a well-conserved DDE catalytic core, confirming CNAG_05910 as a CMC-type transposase encoded within full-length CNEST (Fig S3A). Phylogenetic analysis places CNEST in a distinct clade from CNIRT6, forming part of a strongly-supported clade (Bootstrap value 100%) containing unclassified transposases from diverse animals, including a transposase annotated as an EnSpm/CACTA element in the Pacific oyster (*Crassostrea virginica*) (Fig S3B). This clade is sister to clades of elements containing Chapaev and Mirage transposases from animals, forming a well-supported monophyletic group (Bootstrap value 86%) (Fig S3B). Thus, CNEST encodes a transposase defining a new superfamily within the CMC supergroup that is distinct but most closely related to Chapaev and Mirage superfamilies. In summary, two elements mobilized to mediate drug resistance in case 77: KDZ1, which was detected only at 30°C, and CNEST, which mobilized significantly more frequently at 37°C and is now characterized as a novel active TE in *C. neoformans* belonging to the CMC supergroup. Note that the KDZ superfamily, to which KDZ1 belongs, is completely distinct from the CMC supergroup, which includes CNEST and CNIRT6.

### Diverse TEs drive temperature and strain-specific TE mobilization dynamics in case 7

**Figure 4.**
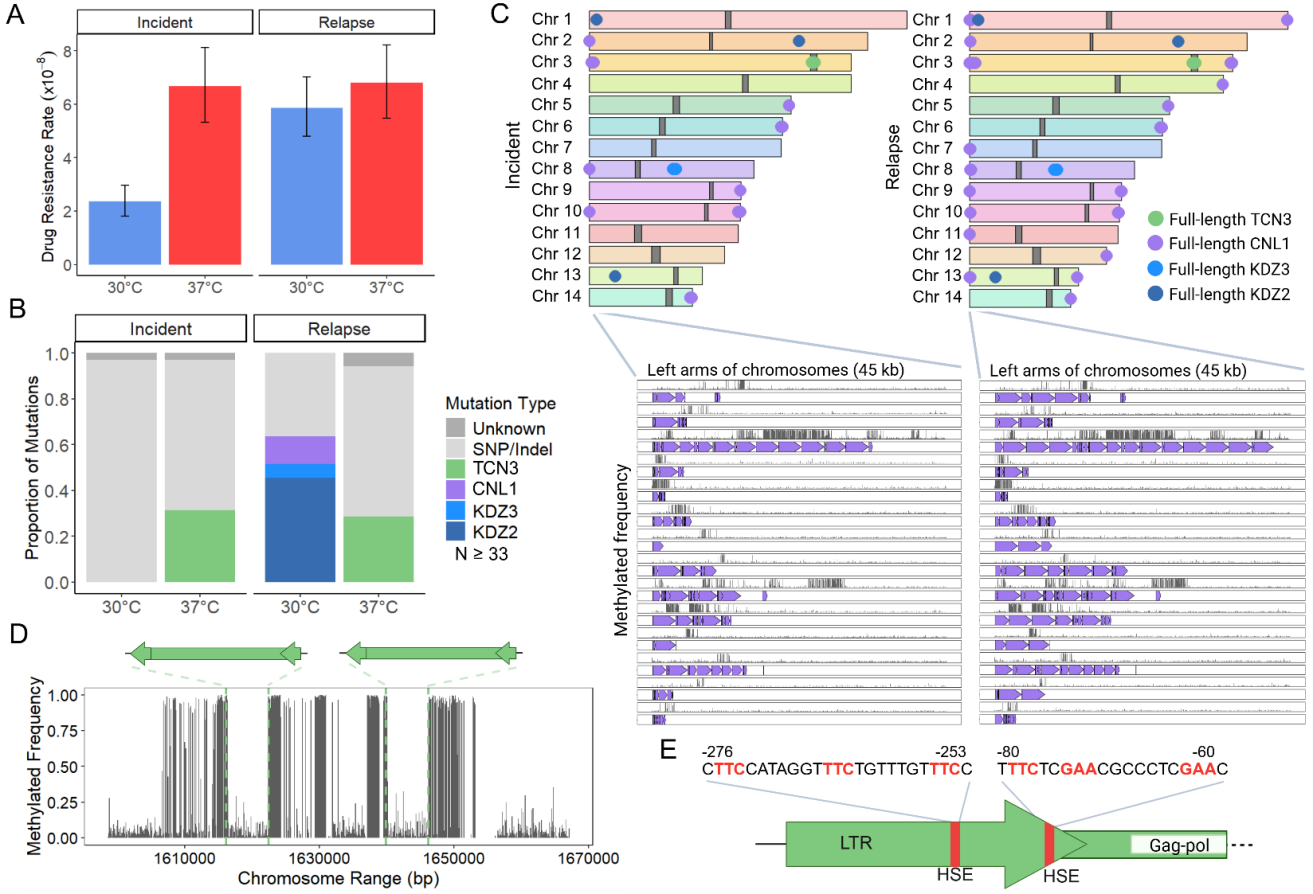
Temperature- and strain-specific TE dynamics in case 7. (**A**) Rap+FK506 drug resistance rates following growth in non-selective YPD medium at 30°C or 37°C. (**B**) Mutation spectra in the *FRR1* gene for independent rap+FK506-resistant mutants. The mutation type “Unknown” indicates failure to amplify *FRR1* or absence of detectable mutations in its sequence (N = 33 – 35). (**C**) Karyograms of the case 7 incident and relapse isolates. Full-length copies of TCN3 (green), CNL1 (purple), KDZ3 (blue), and KDZ2 (dark blue) are shown as circles. Chromosome synteny is indicated by chromosome color. Insets show the 45 kb regions on the left arms of chromosomes in the incident and relapse isolates. Purple arrows indicate all CNL1 BLASTn hits; grey lines represent DNA methylation frequency from long reads, with the y-axis scaled from 0 to 1. (**D**) DNA methylation frequency plotted for the Chr 3 centromere region ±10 kb of the incident isolate. The position and orientation of full-length TCN3 copies is shown. (**E**) Diagram of HSE sequence motifs (red) identified in TCN3 LTRs. Position is shown relative to the gag-pol open reading frame (ORF) translation start site.

The case 7 incident isolate displayed a significantly higher rap+FK506 resistance rate following growth at 37°C compared to 30°C, while the relapse isolate showed no significant difference in drug resistance rates (Fig 4A). For the incident and relapse isolates at 37°C, ~30% of mutations in *FRR1* were caused by insertions of the LTR retrotransposon TCN3, with both full-length and solo LTR insertions detected (Fig 4B, Fig S1C). TCN3 is ~6.3 kb in length with 606 bp LTRs, and insertion into *FRR1* occurred in introns and exons, resulting in 5 bp TSDs of variable sequence (Fig 1C, Fig S6A, B, Table S2). TCN3 is a member of the Ty3/gypsy superfamily that has been previously characterized (54). The TCN3 insertion we identified contains a ~4.7 kb open reading frame with apparently intact Pol domains (reverse transcriptase, RNase H, protease, and integrase) (Fig 1C). Although we did not identify a Gag domain, the first 306 amino acids lack recognizable functional domains and correspond to the region that typically encodes Gag in Ty3/gypsy elements (54). Moreover, the Gag gene is the most variable component of LTR retrotransposons, making it particularly difficult to detect (55). These observations suggest that TCN3 likely represents an autonomous LTR retrotransposon (Fig 1C, Table S2). We recovered no TCN3 insertions at 30°C out of a total of 68 independent mutants screened, suggesting that the element may exclusively mobilize at higher temperatures (Fig 4B).

Surprisingly, over 60% of *FRR1* mutations in the case 7 relapse isolate at 30°C were caused by insertions of diverse TEs, including the non-LTR retrotransposon CNL1 and the DNA transposons KDZ2 and KDZ3 (Fig 4B, Fig S1C). In contrast, mutations in the incident isolate at 30°C were exclusively SNPs/indels (Fig 4B). The longest CNL1 insertion we identified in *FRR1* was ~3.5 kb in length and contained a ~2.5 kb open reading frame with an apparently intact reverse transcriptase domain (Fig 1C, Fig S1C). This maximum insertion length matches previous reports of CNL1 transposition (38). CNL1 insertions identified in *FRR1* ranged from 180 bp to ~3.5 kb likely due to premature termination of reverse transcription which can occur during target primed reverse transcription (TPRT) (56). CNL1 insertions occurred in the *FRR1* 5′ untranslated region (UTR) and in an exon, and were associated with TSDs of variable length (0 – 13 bp), consistent with findings from prior studies (Fig S6A, B) (38). For KDZ2 (~9.2 kb with 139 bp TIRs) and KDZ3 (~10 kb with 169 bp TIRs), insertion into *FRR1* occurred in introns and exons and resulted in 4 – 8 bp TSDs (Fig 1C, Fig S6A, B, Table S2). The internal sequences of KDZ2 and KDZ3 encode putative transposases with KDZ and CxC1 domains (Fig 1C). Although TCN3 and KDZ3 were previously annotated, this study is the first to demonstrate their mobility.

BLAT analysis of case 7 genome assemblies using full-length TE sequences that inserted into *FRR1* as queries revealed conserved copy number and position of full-length TCN3 (2 copies), KDZ2 (2 copies), and KDZ3 (3 copies) between the incident and relapse isolates (Fig 4C). This indicated that the observed KDZ2 and KDZ3 activity in the relapse isolate was not due to an increase in copy number or change in genomic position. Additionally, no DNA methylation was detected on any full-length KDZ2 or KDZ3 copies in case 7 isolates, and no TE sequence polymorphisms were observed between serial isolates (Table S4). We did, however, identify an expansion of CNL1 copies in the relapse isolate, with the number increasing from 16 to 39 full-length copies in arrays in the subtelomeres (Fig 4C). CNL1 copies exhibited varying levels of DNA methylation (Fig 4C, Table S4). The expansion in CNL1 copy number may explain the observed CNL1 mobility in the relapse isolate, which was undetected in the incident isolate (Fig 4B). No genomic rearrangements or other TE-mediated changes, including alterations in copy number or position of TCN3 solo LTRs, were identified between the case 7 isolates.

Interestingly, we recovered 13 total CNL1 insertions in *FRR1*; however, all full-length CNL1 copies in case 7 genome assemblies are located at the ends of incident and relapse chromosome arms, flanked externally by telomeric repeats (“TAACCCCC,” or slight variations thereof), consistent with previous reports (Fig 4C (19,57). To further characterize CNL1, we performed a phylogenetic analysis of the full-length CNL1 insertion in *FRR1* and the CNL1 consensus sequence from Repbase. This analysis revealed that CNL1 is more closely related to non-LTR elements of the R2 superfamily (Fig S3C), which are characterized by a single open reading frame and often insert into specific target sequences (58,59), in contrast to other non-LTR retrotransposons such as LINE1 elements which have weak target site specificity (60,61). CNL1 was found to be most closely related to a telomeric-targeting element, MoTeR, described in the fungal plant pathogen *Magnaporthe oryzae* (Bootstrap value 100%) (62). Prior studies of CNL1 insertions reported that over 70% occurred within other CNL1 copies, but found no strict sequence specificity at insertion sites (19), despite CNL1’s close relationship to sequence-specific elements like MoTeR of the R2 superfamily. However, we did observe similarity in the TSDs between independent CNL1 insertions, suggesting potential sequence-specific target site preferences (Fig S6A, B).

Centromeres in *C. neoformans* are typically enriched for LTR retrotransposons with > 95% of LTR retrotransposons, including TCN3, restricted to centromeric regions in the H99 genome reference strain, though the mechanism or selective pressure driving centromere targeting of insertions remains unclear (39). Although endogenous TCN3 copies in case 7 isolates are restricted to centromeres, 68 independent TCN3 insertions were detected in *FRR1* (Fig 4B). Mapped insertions occurred at variable sequences and positions within *FRR1*, indicating a lack of strong sequence-specific insertion preference (Fig S6A, B).

*C. neoformans* centromeres are also enriched for DNA methylation, which is thought to contribute to repression of TEs present in these regions (40,41). Therefore, we assessed whether the centromeric copies of TCN3 were methylated. If methylated, demethylation and subsequent derepression of TCN3 under heat stress in vitro might explain the observed TCN3 mobility at 37°C in case 7 isolates (63). We found that the full-length copies of TCN3 in the case 7 isolate genomes reside in hypomethylated regions within the predicted centromere region of Chr 3 for the incident and relapse (data for the relapse isolate not shown) (Fig 4D). While five to seven 5mC DNA methylation marks were observed in full-length TCN3 copies, they are located at the end of the 3’ LTR (Fig 4D, Table S4). This region is not typically transcribed, making the observed DNA methylation unlikely to impact TCN3 transposition (64). This suggests that loss of DNA methylation at full-length TCN3 copies is unlikely to underlie the differential activity of TCN3 observed in vitro, where transposition occurred only at 37°C.

Our group previously identified the LTR-retrotransposon TCN12 in *Cryptococcus deneoformans* as heat stress-activated, mobilizing almost exclusively at 37°C (30,31). In *C. neoformans*, heat shock binding elements (HSEs) in the promoter region of heat-responsive genes have been shown to be bound by the transcriptional activator heat shock factor 1 (Hsf1) during heat stress (65). A putative HSE was previously identified in the Tcn12 LTR (31), and upon scanning the TCN3 LTR, we identified two putative HSEs (Fig 4E). This suggests that the elevated mobilization rates of TCN3 and TCN12 at 37°C may result from Hsf1 binding and subsequent upregulation, a hypothesis that our group is currently investigating (65).

### Limited nutrient availability alters TE activity

To better mimic the limited nutrient availability *C. neoformans* experiences in-host and assess impacts on TE activity, we compiled the spectra of mutations in the *FRR1* reporter gene for case 7, 15, and 77 following growth in chemically defined RPMI tissue culture medium at 37°C (66). The medium was supplemented with less glucose (0.2%) compared to YPAD (2%) and had a defined starting pH of 7.0. For five of the six isolates, the same TEs were identified as mobile in YPAD and RPMI medium (Fig S7A, B, C). However, the proportion of TE-mediated insertions during growth in RPMI decreased compared to growth in YPAD at 37°C (Fig S7A, B, C, Fig 2B, Fig 3B, Fig 4B). This indicates that the additional stressor of nutrient limitation may act to suppress TE mobilization in some instances. One exception was the case 15 incident isolate which displayed comparable TE mutation spectra in both growth conditions (Fig S7A, Fig 2B). Additionally, in the case 77 incident isolate, CNL1 was identified inserting into *FRR1*, which had not been previously observed in experiments using YPAD medium (Fig S7B).

We additionally tested the impact of RPMI medium at 30°C for case 7 isolates given the activity of CNL1, KDZ2, and KDZ3 observed in the relapse isolate at 30°C in YPAD medium (Fig 4B). Interestingly, the use of RPMI medium altered TE dynamics in an isolate- and temperature-specific manner in case 7 isolates (Fig S7C). Differences between YPAD and RPMI medium include: (1) no TCN3 insertions observed in the relapse at 37°C, (2) KDZ3 insertions caused ~35% of mutations in the incident at 30°C, which were absent in YPAD, and (3) variations in the proportion of CNL1, KDZ2, and KDZ3 insertions at 30°C and 37°C in the relapse, highlighting the complex relationship between environment, genetic background and TE activity in case 7 isolates.

### CNEST heat stress-activation is conserved in a geographically and temporally distinct environmental isolate

**Figure 5.**
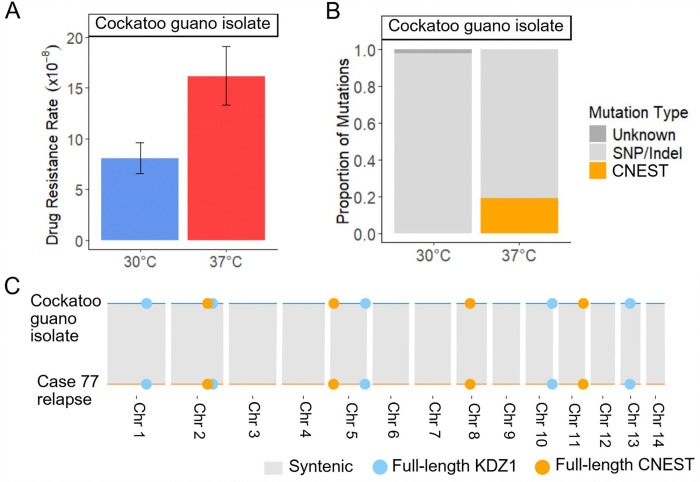
Heat stress-induced CNEST activation is conserved in an environmental isolate. (**A**) Rap+FK506 drug resistance rates following growth in non-selective YPD medium at 30°C or 37°C. (**B**) Mutation spectra in the *FRR1* gene for independent rap+FK506-resistant mutants. The mutation type “Unknown” indicates failure to amplify *FRR1* or absence of detectable mutations in its sequence (N = 42 – 52). (**C**) Chromosome-level synteny between the cockatoo guano and case 77 relapse isolates. Full-length copies of CNEST (yellow) and KDZ1 (light blue) are shown as circles.

BLAT analysis revealed full-length CNEST and KDZ1 copies in a previously published *C. neoformans* genome, sequenced from an environmental isolate collected from cockatoo droppings in Boston in 2000 (designated cockatoo guano isolate) (26,67). To better understand mobility of CNEST and KDZ1 in a different strain background, we assessed rap+FK506 drug resistance rates and the spectra of mutations in *FRR1*. Indeed, cockatoo guano isolate drug resistance rates were significantly elevated during growth at 37°C compared to 30°C (Fig 5A). Approximately 20% of mutations in *FRR1* at 37°C were CNEST insertions, while no CNEST insertions were observed at 30°C, strongly supporting that CNEST transposition is induced by heat stress in the cockatoo guano isolate (Fig 5B). The cockatoo guano and case 77 isolates both belong to the VNII clade and are geographically and temporally distinct, as the case 77 isolates were collected eight years later in South Africa. To assess the copy number and genomic context of CNEST and KDZ1, we performed BLAT searches and synteny analysis using SyRI, which demonstrated that both the copy number and genomic position of CNEST and KDZ1 were conserved (Fig 5C). This may suggest that mobilization of CNEST and KDZ1 in the VNII lineage is infrequent, that mobilization events are typically detrimental to fitness, or that there is selective pressure to maintain both the copy number and genomic context of these elements.

A recent study performed two rounds of passaging the cockatoo guano isolate through A/J mice via retro-orbital intravenous injection, collecting isolates from the brain and lungs following each passage (26). Additionally, the cockatoo guano isolate was the point-source isolate for a case of cryptococcal meningitis, from which a single isolate was collected from the patient (PU isolate) (67). To assess whether transposition of CNEST or KDZ1 occurred during murine passage or human infection, Southern analysis was performed using 551 bp CNEST- and 567 bp KDZ1-specific probes, along with restriction enzymes that cut once within the TE sequence, outside of the probe site, to produce a range of fragment lengths (Fig S8A). The same hybridization band patterns were observed in the original cockatoo guano isolate and in all isolates recovered following infection (Fig S8B, C), indicating that no CNEST or KDZ1 mobilization occurred during infection, despite detection of CNEST transposition in vitro at 37°C (Fig 5B). Thus, while heat-stress activation of CNEST appears conserved between environmental and clinical isolates, no transposition events were detected in serial isolates recovered from human or murine infections.

### Full-length copies of TEs are present across multiple isolates and lineages

Because TE mobility was highly isolate- and strain-dependent in the case 15, 77, and 7 isolates, where the TEs captured mobilizing were unique to each case, we hypothesized that this may be due to the absence of full-length copies in certain isolate backgrounds. To evaluate the prevalence of full-length TE copies across *C. neoformans* isolates, we conducted homology searches in assembled genomes using the active TE sequences that were identified inserting into the *FRR1* gene as queries. Full-length copies were defined as hits with ≥ 90% nucleotide identity and ≥ 95% coverage of the query sequence. We identified full-length copies of CNIRT6, CNEST, TCN3, KDZ1, and KDZ3 in several genomes of isolates where transposition was not detected using the *FRR1* reporter screen. We did not, however, identify full-length copies of KDZ2 or CNL1 in other isolates; these elements were present only in those where transposition occurred (Fig S9A). For example, at least one full-length copy of CNEST was found in all genomes assessed, including strains from the VNI, VNII, and VNBII lineages (Fig S9A).

To assess whether full-length copies were present in the 15 *C. neoformans* cases initially screened without available whole-genome assemblies, we performed TE copy number estimation by aligning short reads to TE sequences and estimating copy number based on normalized read depth (defined as the depth at which ≥ 95% of the TE sequence is covered) (32). This analysis revealed that full-length copies of potentially active elements were likely present even in isolates in which no TE mobilization was detected in the initial screen, including cases where estimated copy numbers exceeded those in isolates with observed mobilization (Fig S9B).

### DNA methylation status may explain differential mobility of TCN3

Although each TE identified in this study was detected mobilizing exclusively in a single isolate background, with the exception of CNEST and CNL1, additional full-length copies were found in other assemblies or inferred through copy number estimation (Fig S9A, B). This suggests that TE activity may be differentially regulated across isolate backgrounds. For example, elevated TE mobility in some isolates may be explained by a loss of RNAi functionality or the absence of DNA methylation on TE sequences.

To explore potential regulatory mechanisms, we first assessed the levels of DNA methylation in full-length TE copies in which mobilization was either observed or not observed using the *FRR1* reporter gene. Full-length TCN3 copies were highly methylated in cases 15 and 1, whereas hypomethylated copies were observed in case 7 isolates (Table S4). In contrast, DNA methylation was generally absent from full-length DNA transposons in strains where no mobilization was observed. For example, in whole-genome assemblies, full-length KDZ1, KDZ3, and CNEST elements were unmethylated in isolates where transposition was not observed in *FRR1*, with the exception of CNEST in case 1 (Table S4). These findings suggest that while DNA methylation may play a role in regulating TCN3 mobility at the centromeres, the methylation status of full-length DNA transposons (which are primarily unmethylated) does not explain isolate- or strain-specific differences in mobility.

Next, we examined whether genes required for RNAi function were intact in cases where TE mobility was observed. In cases 7, 15, 77, and the cockatoo guano isolate, we screened for predicted loss-of-function mutations (defined as predicted protein sequences < 85% of the expected length) using a previously published list of genes essential for RNAi (32). No genes required for RNAi function exhibited truncated protein sequences, providing no evidence for loss-of-function mutations. However, it is possible that nonsynonymous changes could also render RNAi ineffective, which is a limitation of this approach. Alternatively, differential targeting by RNAi or the accumulation of point mutations in TEs may have rendered some full-length copies incapable of transposition.

### DNA transposons are predominantly unmethylated and commonly found in gene-rich regions

**Figure 6.**
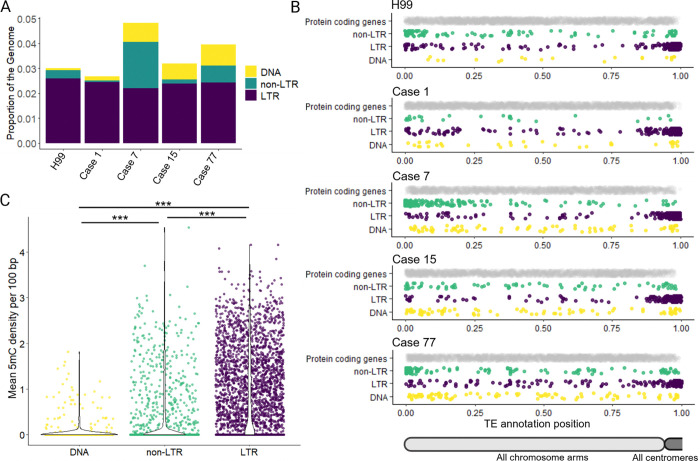
Genomic distribution and DNA methylation differ among non-LTR, LTR, and DNA transposons. (**A**) The proportion of the genome composed of non-LTR, LTR, and DNA transposons, based on the Earl Grey TE annotation. (**B**) Scaled positions of protein coding genes and non-LTR, LTR, and DNA transposons across chromosome arms. Chromosomes were split at the midpoint of the centromere region, and protein-coding gene and TE positions were scaled from 0 to 1 based on chromosome arm length. Data for all chromosome arms is plotted. (**C**) Mean 5mC DNA methylation density per 100 bp for DNA, non-LTR, and LTR TEs in isolates from cases 1, 7, 15, and 77. Violin plots are overlaid to illustrate the distribution. Significance was assessed using the pairwise Wilcoxon rank-sum test with continuity correction. *** denotes Bonferroni-adjusted *p* < 2e-16. The first genome assembly from serially collected clinical isolates was chosen as a representative for all cases.

We observed that all full-length, active DNA transposons mapped in the case 15, 77, and 7 genomes were located in gene-rich regions along the chromosome arms (Fig 2D, 3D, 4C). Previous studies have highlighted distribution biases of LTR and non-LTR retrotransposons in *Cryptococcus*, with LTR elements predominantly localized in centromeres (39), and non-LTR elements enriched at chromosome ends (38). To further investigate the genomic distribution of DNA transposons in *C. neoformans*, which has not previously been described, we took a comprehensive, unbiased approach to assess genome-wide TE content and distribution. De novo TE annotation was performed using the Earl Grey package (68) for cases 1, 7, 15, and 77, as well as the genome of the reference strain H99, in which TE mobilization has not been observed unless RNA interference (RNAi) is disrupted (36,38). Results from the first assemblies of serially collected isolates are shown, as assemblies of later relapse isolates from the same patients yielded similar results. TEs occupied approximately 3 – 5% of the genomes analyzed (Fig 6A). While the proportion of LTR-retrotransposons remained relatively consistent (2.2 – 2.6%), greater (more than tenfold) variation was observed in the proportion of DNA transposons (0.08 – 0.85%), and non-LTR retrotransposons (0.06 – 2.06%) (Fig 6A). Notably, the *C. neoformans* reference strain H99 had the lowest proportion of DNA transposons among all isolates analyzed.

To visualize TE distributions, chromosomes were split at the midpoint of the predicted centromere. For each chromosome arm, the length, TE positions, and protein-coding gene annotation positions were scaled from 0 to 1, allowing data from all chromosome arms to be plotted on a single track. In agreement with previously published data, LTR and non-LTR elements cluster in the predicted centromeres and subtelomeres, respectively; these are regions with a lower density of protein-coding genes (Fig 6B). By contrast, DNA transposons appear to be more evenly distributed across chromosome arms (Fig 6B). We used quantile-quantile (QQ) plots to assess whether the positions of DNA transposons along chromosome arms follow a uniform distribution. For each genome, we generated theoretical quantiles as sorted random numbers between 0 and 1; the total number of theoretical quantiles matched the total number of DNA transposons. The plotted points fell approximately along the diagonal of the QQ plots, indicating that DNA transposons were distributed roughly uniformly across the genome (Fig S10).

To assess how frequently DNA transposons were methylated, given their presence in gene-rich regions that are not typically methylated in *C. neoformans* (41,42), we compared their 5-methylcytosine (5mC) DNA methylation levels to those of LTR and non-LTR elements. First, we assessed average methylation density per 100 bp. DNA transposons exhibited significantly lower average 5mC densities compared to both non-LTR and LTR retrotransposons (pairwise Wilcoxon rank-sum test with continuity correction; adjusted *p* values < 2e-16 for both comparisons) (Fig 6C). Additionally, LTR elements exhibited significantly higher DNA methylation densities compared to non-LTR elements (pairwise Wilcoxon rank-sum test with continuity correction; adjusted *p* value < 2e-16). Second, we assessed the proportion of TE annotations per genome containing at least one high-confidence 5mC call (methylated frequency from nanopore long-read signal data > 0.75). According to this analysis, we found that LTR elements are by far the most consistently methylated (55.6 – 71.2% of LTR retrotransposons contained at least one predicted 5mC), followed by non-LTR elements (7.7 – 53.4%), and DNA transposons (5.4 – 15.5%) (Fig S11A, B). The number of methylated LTR and non-LTR retrotransposons was significantly enriched compared to DNA transposons (Fisher’s method combined *p* values: 3.2e-64 for LTR elements and 9.2e-19 for non-LTR elements).

To estimate background levels of DNA methylation in protein-coding genes, we lifted over gene annotations from the H99 reference genome. The percentage of protein-coding genes with at least one high-confidence 5mC call ranged from 0.5 – 4.5%, indicating that DNA TEs remain significantly enriched for DNA methylation relative to endogenous protein-coding genes (Fisher’s method combined *p* value 3.9e-10), albeit at a lower frequency than LTR and non-LTR elements. Thus, the majority of DNA transposons across all genomes analyzed are unmethylated, and those that are methylated exhibit a lower methylation density compared to non-LTR and LTR retrotransposons (Fig 6C, Fig S11A, B). This suggests that DNA methylation is unlikely to play a major role in modulating the activity of DNA transposons in *C. neoformans*.

## DISCUSSION

Understanding the interplay between mobile genetic elements, environmental stressors, epigenetics, and genetic background is critical for uncovering factors that influence evolutionary rates and the capacity for adaptive change, particularly in the context of fungal pathogens. In this study, we screened a collection of clinical *Cryptococcus* isolates for transposable element (TE) insertions in the *FRR1* reporter gene and profiled rates of rapamycin and FK506 (rap+FK506) resistance, as well as mutation spectra, at both 30°C and the heat-stress condition of 37°C. We also generated telomere-to-telomere genome assemblies to assess TE copy number, genomic position, and DNA methylation status in isolates from patients with recurrent cryptococcal infections.

### *C. neoformans* hosts diverse active transposable elements

We identified seven TE families mobilizing to confer rap+FK506 resistance, including both class I retrotransposons (LTR and non-LTR) and class II DNA transposons (Fig 1). Several of these elements, such as TCN3, CNIRT6, and KDZ3, were not previously recognized as active. In addition, we discovered a novel, active, and previously uncharacterized element, which we named CNEST. The diversity of active TEs in *C. neoformans* stands in striking contrast to other yeasts commonly used to study TEs, such as *Saccharomyces cerevisiae* and *Schizosaccharomyces pombe*, which harbor only LTR retrotransposons (69,70). In these species, TE mobilization studies typically rely on reporter constructs driven by inducible heterologous promoters, due in part to the low de novo transposition rates of endogenous TEs (71–74). By contrast, in *C. neoformans*, screening for mutations at a single genomic locus using a ‘transposon trap assay’ frequently revealed insertions of endogenous TE copies with diverse transposition mechanisms (Figs 2 – 5). The ability to readily capture multiple active TE families mobilizing within a single *C. neoformans* strain underscores the potential for utilizing forward mutation reporters as a powerful, genetically tractable model for studying TE mobilization strategies and regulation.

Phylogenetic analysis of transposases from the DNA transposons CNIRT6 and CNEST revealed that they belong to the CMC supergroup first described by Yuan and Wessler (2011) (Fig S3). This supergroup includes EnSpm, one of the two transposon systems originally discovered in maize by Barbara McClintock (51,75). However, CNIRT6 and CNEST are only distantly related and fall within two monophyletic clades among CMC elements: one containing CNIRT6 and transposase sequences from plants, animals, and fungi, and a second containing CNEST and transposases from animals and fungi. Thus, *C. neoformans* hosts active members of two different CMC superfamilies, a notable finding given that this group of DNA transposons remains largely understudied in fungi and animals. CNEST and CNIRT6 offer an opportunity to study two distinct active CMC transposons in a model fungal species.

Notably, the use of a single reporter gene to capture TE insertions may have limited our potential for active mobile element discovery, since some TEs have preferences for specific insertion sequences or genetic compartments (10). However, the *FRR1* reporter system recovered insertions of TCN3 and CNL1, which appear to target centromeric and subtelomeric regions, respectively. TCN3 insertions in *FRR1* occurred at multiple positions and were associated with variable terminal site duplications (TSDs), suggesting weak sequence specificity (Fig S6). Alternatively, TCN3 may recognize non-sequence-based features of centromeres, such as centromere-specific histone variants (79) or heterochromatin-associated proteins (80), or its insertion pattern may be shaped by selective pressures. Since our approach captures insertions only at the *FRR1* locus, we cannot assess genome-wide insertion preferences. It remains possible that insertion into *FRR1* is relatively rare and that most de novo TCN3 insertions occur within centromeric regions, warranting further investigation in future studies.

CNL1 copies are enriched in subtelomeric regions in case 7 isolates and other *C. neoformans* genomes (38); however, we also recovered insertions of CNL1 within *FRR1* (Fig 4). Phylogenetic analysis revealed that CNL1 is most closely related to R2 elements, and specifically to MoTeR, a telomere-targeting element characterized in the fungal plant pathogen *Magnaporthe oryzae* (62). Although prior studies have reported that CNL1 preferentially inserts into copies of itself within arrays near telomeric repeats, its precise sequence specificity remains undefined. Through limited mapping, we observed two insertion locations for CNL1 within *FRR1*: one in the 5′ UTR and another within an exon (Fig S6). These insertions generated TSDs of variable length, though interpretation was complicated by premature termination of target primed reverse transcription (TPRT) at different positions within the element. Notably, many of the observed TSDs contained the sequence ‘CACCCCT,’ which resembles the *C. neoformans* telomere seed sequence (‘AACCCCCT’ or similar variants). Further work is needed to clarify CNL1’s target site specificity, including the extent to which it targets telomeric repeats like MoTeR, inserts into its own sequence, or both, and to understand how these insertion preferences contribute to its transposition into *FRR1*. Together with findings from TCN3, these observations highlight important open questions about the factors that govern target site selection and the evolutionary forces shaping TE insertion patterns across genomic compartments. Intriguingly, one such factor may be stress, which has been shown to alter target specificity. For example, Ty5 phosphorylation is regulated by nutrient deprivation stressors, shifting its insertion profile from heterochromatic regions to more broadly distributed sites across the genome (80).

Lastly, most observed TE mobilization events were driven by apparently full-length TE copies containing detectable functional domains required for mobilization. Phylogenetic analysis revealed full-length, seemingly intact transposase sequences within mobilized DNA transposons, and functional domains required for mobilization were identified in mobilized TCN3 and CNL1. We found no evidence for the mobilization of non-autonomous TE copies (significantly shortened or lacking apparent mobilization machinery). However, we did detect truncated CNL1 insertions, likely resulting from premature termination of reverse transcription, as well as instances of solo TCN3 LTRs, which likely arose from non-allelic recombination between LTR sequences following TCN3 insertion. This contrasts with other organisms, such as humans, plants, and *Drosophila*, where shortened non-autonomous TEs frequently transpose (76–78).

Collectively, we identified seven mobilizing TE families: DNA transposons from the KDZ superfamily and CMC supergroup, alongside a Ty3/Gypsy LTR element and an R2 non-LTR element actively transposing in *C. neoformans*. These TEs employ distinct transposition mechanisms and show preferences for different genomic compartments, revealing a rich “ecosystem” of active transposable elements within *C. neoformans*.

### Transposable element mobilization in *C. neoformans* is strongly influenced by temperature

Previous work in *C. deneoformans* demonstrated that heat stress increases TE mobilization (30,31). Building on this, we screened 15 clinical *C. neoformans* isolates for TE insertions in the *FRR1* reporter gene at 37°C. While some elements followed the pattern previously observed in *C. deneoformans*, mobilizing more frequently at elevated temperature, we also uncovered TEs that behave differently. Several elements (CNL1, KDZ1, KDZ3) were found to mobilize exclusively at 30°C. Others (CNIRT6, CNEST) mobilized at both temperatures, but with higher frequency at 37°C. Therefore, by limiting our initial screen to 37°C and examining only ten independent mutants, we may have overlooked strains harboring mobile elements that are active only at lower temperatures or that mobilize at low frequency.

The LTR retrotransposon TCN3 was observed to mobilize exclusively at 37°C (Fig 4), paralleling earlier findings in *C. deneoformans*, where heat stress activated the LTR retrotransposon TCN12, which contains a heat shock element (HSE) in its LTR promoter region (31). In TCN3, we identified two putative HSEs within its LTR, suggesting potential regulation by the heat shock transcription factor Hsf1, a key coordinator of the heat shock response (65). While heat shock-induced TE activation via host machinery has been demonstrated in plants and animals (81–83), this mechanism has not been described in fungi. We are currently testing the hypothesis that TCN3 and TCN12 co-opt the host heat shock response to facilitate their mobilization.

Other temperature-dependent effects on TE mobility may be attributed to self-regulatory mechanisms of the TE-encoded machinery or to alteration of TE suppression mechanisms at different temperatures. As an example of the former, Ty1 element mobilization in *S. cerevisiae* is most frequent at 22°C and is not observed at temperatures above 34°C due to inactivity of the Ty1-encoded protease (84). As an example of the latter, in *Drosophila*, heat stress triggers heat shock protein 70 (Hsp70) to displace PIWI-interacting RNA (piRNA) biogenesis factors to the lysosome for degradation, leading to transcriptional activation of TEs (85). In *C. neoformans*, a recent study found a link between the Calcineurin signaling pathway, which is necessary for stress adaptation and growth at 37°C, and RNAi-mediated silencing of a repetitive transgene, suggesting a potential mechanism for altered RNAi function during heat stress (86). Further studies are needed to elucidate the mechanisms underlying differential TE mobilization under heat stress in *C. neoformans*, which will inform our understanding of TE dynamics and their role in adaptive evolution during infection and in other heat-stress contexts.

While temperature plays a significant role in driving TE mobilization, additional environmental factors likely contribute as well. Nutrient limitation in RPMI medium altered TE mobilization dynamics across most strains (Fig S7). Moreover, although CNL1 mobilization was only observed in vitro at 30°C, it was the sole TE implicated in genomic changes during recurrent cryptococcal infection, where cells encounter mammalian body temperature. This underscores the complex interplay between environmental conditions and TE activity, highlighting that temperature is not the sole regulator. Other stressors or environmental stimuli may act concurrently to activate or repress TE mobility, although under our in vitro conditions, temperature remains a major regulator of TE mobilization across genetically diverse *C. neoformans* strains.

### DNA transposons are hypomethylated relative to retrotransposons, and are commonly found in gene-rich regions

Given that *C. neoformans* lacks a de novo cytosine DNA methyltransferase, DNA methylation as a TE suppression mechanism is relatively inflexible and unlikely to target newly inserted or unmethylated TE sequences (41). Despite this, a substantial proportion of TEs exhibit DNA methylation: 55.6 – 71.2% of LTR elements and 7.7 – 53.4% of non-LTR elements (Fig S11). In contrast, DNA transposons are less frequently methylated, with only 5.4 – 15.5% showing methylation, and when methylated, they exhibit significantly lower average 5mC density compared to LTR and non-LTR elements (Fig 6). This reduced level of methylation may contribute to the more frequent mobilization of DNA transposons observed in this study, where five of the seven mobile elements identified were DNA TEs. Furthermore, full-length DNA transposons are more evenly distributed across the genome and are commonly found within gene-rich regions, whereas LTR and non-LTR retrotransposons tend to be enriched in gene-poor centromeric and subtelomeric regions. As a result, DNA TEs may be more likely to mediate genetic changes that alter the expression or function of protein-coding genes which could drive adaptation under stress conditions.

### Transposable elements provide a mechanism for rapid genetic change and facilitate adaptation in *C. neoformans*

Growth at mammalian body temperature (37 °C) significantly increased the rate of acquired resistance to rap+FK506 in both clinical and environmental isolates (Figs 2–5). In all cases, this effect was driven by elevated TE insertion rates, highlighting the role of TEs in generating adaptive phenotypes such as antifungal resistance. These findings suggest that in-host conditions or other heat stress contexts may enhance evolvability within *C. neoformans* populations through bursts of rapid TE mobilization.

RNA interference (RNAi) is the primary known mechanism for TE suppression in *C. neoformans*, and loss of RNAi function is associated with hypermutator phenotypes driven by uncontrolled TE activity (38). However, recent work has shown that this is not always the case, as some isolates with loss-of-function mutations in essential RNAi genes maintain low TE burdens and mutation rates (32). In this study, none of the clinical or environmental isolates profiled for mutation spectra harbored predicted loss-of-function mutations in RNAi pathway genes. Nevertheless, isolates from cases 15 and 77 exhibited rap+FK506 resistance rates at 37°C comparable to those observed in hypermutator strains, including the mismatch repair-deficient *msh2Δ* mutant (32,38). This suggests that, in certain strain backgrounds, heat stress alone can be sufficient to induce a hypermutator-like phenotype through increased TE activity, even in strains with a relatively low TE burden (e.g., two full-length copies of CNIRT6 in case 15 isolates).

When assessing the impact of TEs on in-host microevolution, we observed, in one notable case, an expansion of CNL1 copy number in serial isolates collected from a patient with recurrent cryptococcal meningitis, suggesting a role for TE activity in within-host evolution (Fig 4). However, no transposition or TE-mediated genomic changes were detected in other longitudinally sampled clinical isolates or in isolates collected following murine passaging. This may suggest that TE mobilization events are rare in vivo, that TE-mediated changes did not confer a selective in-host growth advantage during infection, or that TE-mediated changes occurred in subpopulations not captured by sampling a single isolate. Previous studies in *C. deneoformans* found evidence of transposition occurring during murine infection (30,31). In these cases, however, only drug-resistant isolates were selected for recovery from the mice, requiring that loss-of-function mutations at specific reporter loci had occurred in the recovered isolates. In contrast, there was no selective pressure for evolved drug resistance or other traits during the recovery of patient and mice isolates examined in this study.

### Genetic background influences TE mobility

Telomere-to-telomere genome assemblies revealed that the presence of full-length TEs does not reliably predict their mobilization in vitro. For instance, in clonally related case 7 isolates, we observed striking differences in KDZ2 and KDZ3 mobilization at 30°C, despite these elements being present at identical copy number and genomic positions, with identical sequences, and lacking DNA methylation in both isolates. Interestingly, of the two single nucleotide polymorphisms (SNPs) previously identified between case 7 isolates, one was an amino acid substitution in *SET5* (CNAG_03890), a predicted protein lysine methyltransferase (24). This suggests that alternative epigenetic regulation may be driving differential mobility, a hypothesis we plan to investigate further. Similarly, unmethylated full-length CNEST copies were identified in all assembled genomes except for case 1; however, mobilization was observed only in the case 77 and cockatoo guano isolates. These observations suggest that isolate- and strain-specific TE activity may be regulated by differential RNAi targeting, alternative suppression mechanisms beyond DNA methylation, or disabling mutations. Future studies are needed to dissect the factors underlying differential TE mobility, which may include TE copy number polymorphisms, sequence variation within TEs, or genetic variation in host factors that control transposition.

Altogether, our findings highlight the complex interplay between genetic background, epigenetics, and environmental conditions in governing TE mobilization, with temperature emerging as a major determinant. Further studies should investigate the extent to which stress-induced transposition generates genetic diversity and whether this diversification drives adaptation to infection-relevant stressors and the repeated emergence of antifungal drug resistance in *C. neoformans*.

## METHODS

### Strains and growth media

The strains used in this study are listed in Table S1. Strains were grown in YPAD medium (1% yeast extract, 2% peptone, 2% dextrose, 0.025% adenine) with 2% agar added for solid media. For nutrient limiting conditions, strains were cultured on RPMI plates containing 3.45% MOPS, 0.2% glucose, 2% agar, and 1.04% RPMI 1640 powder (Sigma #R1383). Mutants resistant to rapamycin and FK506 (rap+FK506) were selected for on YPAD plates supplemented with 1 μg/mL rapamycin (LC Laboratories) and 1 μg/mL FK506 (Astellas).

### Fluctuation assay and mutation spectra

Cultures were inoculated with independent colonies; each colony was split between a 30°C and a 37°C culture and grown for 3 days in 1 mL of liquid YPAD, rotating at either 30°C or 37°C. Cells were pelleted, washed, and resuspended in water. Cells were appropriately diluted, plated on solid YPAD medium, and incubated at 30°C for 3 days. Colony counts were used to calculate colony forming units (CFUs) per culture. The remainder of the culture was plated on two rap+FK506 plates and incubated at 37°C for four days before counting colonies. Total CFUs and rap+FK506-resistant colony counts were used to calculate mutation rates using the Fluctuation AnaLysis CalculatOR (FALCOR), applying the Ma-Sandri-Sarkar (MSS) Maximum Likelihood Method (87). A single rap+FK506-resistant colony from each culture was selected for genomic DNA isolation, performed using either in-house reagents or a MasterPure Yeast DNA Purification Kit (VWR #76081–694).

The *FRR1* gene was amplified for Sanger (Azenta) or linear amplicon long-read sequencing (Plasmidsaurus). Amplification of *FRR1* was carried out using a variety of polymerases: MyTaq Mix (Thomas Scientific #C755G91), Apex Taq RED Master Mix (Genesee Scientific #42–138), Platinum SuperFi II PCR Master Mix (ThermoFisher #12368010), Q5 High-Fidelity DNA Polymerase (NEB #M0491S), and LongAmp Taq DNA Polymerase (NEB #M0323S). We often observed “phantom” wild-type–sized secondary bands when amplifying across TEs, which can occur as a PCR artifact when amplifying across long repetitive DNA sequences (88). In these instances, gel extraction using the Zymoclean Gel DNA Recovery Kit (Zymo Research #D4007) was performed on the higher molecular weight bands, which were then sent for sequencing. For TEs identified as inserting into *FRR1*, where the full sequence was obtained by linear amplicon sequencing, junction PCR with primers targeting the internal TE sequence from the same isolate was used to confirm a subset of insertion events. Primers used to amplify *FRR1* and for TE-specific junction PCR are listed in Table S5.

Sanger sequencing data were analyzed in SeqMan Ultra 17 or DNASTAR Navigator 17 by aligning reads to the wild-type *FRR1* sequence for each isolate. TEs were assigned to families based on the 80–80-80 rule: nucleotide similarity greater than 80%, over more than 80 base pairs, covering at least 80% of the recovered sequence length (48). The TE consensus sequence for TCN3 was obtained from Repbase. For TEs lacking family-level consensus sequences, GenBank submissions were used to assign family membership (GenBank accessions: PQ181658 [KDZ1], PQ181660 [KDZ2], and PQ181661 [KDZ3]). CNL1 family membership was called based on sequence similarity to CNL1 mapped by Priest et al. (2022) in Bt65 and Bt81 genome assemblies (BioProject accession PRJNA749953).

### RPMI mutation spectra

To generate independent mutants during growth on RPMI medium, a prong-plating procedure was carried out. Single colonies were inoculated in liquid YPAD and grown overnight rotating at 30°C, then cells were washed and diluted in water. A custom tool containing 121 flat-tipped pins was used to transfer approximately ~10^4^ cells/pin onto non-selective RPMI plates (89). To confirm there were no pre-existing mutations, cells were also spotted on rap+FK506 plates. Confluent spots of cells on these plates would indicate mutations occurred during the overnight growth in YPAD and prong plating would not be possible to generate independent mutants. If less than 5 colonies appeared on these control plates, experiments were continued. RPMI plates were incubated for 3 days at either 30°C or 37°C. RPMI plates were then replica plated to rap+FK506 plates and incubated at 37° for 3 days. A single colony was picked from each spot for genomic DNA isolation, followed by *FRR1* amplification and either Sanger sequencing (Azenta) or junction PCR. *FRR1* amplification, junction PCR, Sanger sequencing data analysis, and TE classification were carried out as described above.

### TE insertion rate

TE insertion rate was calculated using the proportion of independent mutants with a given TE insertion multiplied by the drug resistance rate. The 95% confidence intervals (CI) for proportions were determined using the vassarstats.net proportion CI calculator including continuity correction. 95% CI for TE insertion rates were determined by a method employing the square root of the sum of the squares (using the component 95% CI for rates and proportions) (90).

### Transposase and reverse transcriptase protein sequence alignments and phylogenies

Transposase and reverse transcriptase protein sequences were obtained from Yuan and Wessler (2011) for CMC (CACTA, Mirage, Chapaev) related sequences, Repbase consensus sequences (https://www.girinst.org/), and from BLASTp (for translated mRNA sequences) or tBLASTn (for translated genomic sequences) via NCBI (https://blast.ncbi.nlm.nih.gov/Blast.cgi). Protein sequences were aligned using MAFFT (https://www.ebi.ac.uk/jdispatcher/msa/mafft) with the following parameters, protein matrix = BLOSUM62, gap opening penalty = 1.53, gap extension penalty = 0.123, tree rebulding number = 2, max iterations = 2, and FFTS = none. Aligned protein sequences were then used to construct maximum likelihood phylogenies with 100 bootstrap replicates using MEGA version 11 (91).

### Nanopore sequencing

To extract high molecular weight DNA for library preparation, overnight cultures were grown in 50 mL liquid YPAD shaking at 30°C. Cell were pelleted and lyophilized overnight. Genomic DNA was extracted using a cetyltrimethylammonium bromide (CTAB) extraction method with some modifications (92). Briefly, cells were lysed by bead beating, and DNA extraction buffer (100 mM Tris-HCl, pH 7.5; 1% w/v CTAB; 0.7 M NaCl; 10 mM EDTA; 1% v/v 2-mercaptoethanol) was added and incubated at 65°C for 30 minutes. Chloroform purification was then performed, and layers were separated by centrifugation. The aqueous layer was isolated, and DNA was subsequently precipitated, washed, and treated with RNase A. Sodium acetate (NaOAc) was then added to a final concentration of 0.6 M, and chloroform purification was repeated. DNA was precipitated again, washed, and spooled to collect high molecular weight DNA.

DNA quality was assessed using NanoDrop One A260/A280 and A260/A230 ratios (Thermo Fisher Scientific) and quantified using Qubit dsDNA Broad Range Assay Kit (Invitrogen #Q32850) and the Qubit 4 Fluorometer (Invitrogen). To assess DNA fragment length, clamped homogeneous electric field (CHEF) gel electrophoresis was performed using the CHEF Mapper (Bio-Rad). Libraries were prepared using the SQK-LSK110 or SQK-NBD114.24 and EXP-NBD104 kits and sequenced using the MinION Mk1C following manufacturer’s instructions (Oxford Nanopore). Sequencing was performed using the MinKNOW software at the default voltage for at least 48 hours. Basecalling was performed using the latest Guppy software (v7.1.4, v6.3.8, or v6.4.8), or with Dorado (v0.8.1). The specific sequencing kit, flow cell, and basecalling methodology used for each isolate are listed in Table S3.

### Genome assembly

Demultiplexing was performed using Guppy (v6.5.7). The Canu (v2.2) genome assembler was used to assemble long reads into contigs, small contigs likely representing mtDNA or contaminants (< 200 kb) were discarded (93). For the case 15 incident isolate only, assembly was performed by combining outputs from Canu and Flye (v2.9.5) (94). Genome polishing was performed using long reads generated in this study and short reads available under the SRA umbrella project PRJNA37160 (24). Long reads were mapped to contigs using Minimap2 (v2.24) followed by Medaka (v1.5.0 or v2.0.1) polishing using the appropriate model (95). Short reads were mapped to the draft assembly using Bowtie2 (v2.4.4) followed by Pilon (v1.24) polishing, which was performed in five iterations, re-mapping the short reads to each new draft assembly before the next iteration of polishing (96,97). Canu-corrected Nanopore reads were mapped to the assembly using Minimap2 (v2.24) and coverage was visually inspected in the Integrative Genome Viewer (IGV) (98). To confirm generation of telomere-to-telomere assemblies, contig ends were inspected for the presence of telomere seed sequences (99). Assembly breaks at the rDNA locus were artificially merged and separated by 50 Ns. Contigs were oriented to match the first serially collected isolate assembly to produce final nuclear genome assemblies. For each isolate, Canu parameters, polishing specifications, and assembly statistics are provided in Table S3.

### DNA methylation detection

5-methylcytosine (5mC) DNA methylation at CpG sites was called using Nanopolish (v0.14.0) or f5c (v1.5) (see Table S3 for isolate-specific details). Minimap2 (v2.24) was used to map long reads to the final nuclear assembly for each isolate. For DNA methylation detection using f5c, POD5 files were converted to BLOW5 using blue-crab (v0.2.0). The Nanopolish helper script methylation_frequency.py was used to calculate the methylation frequency for CpG sites.

### Centromere mapping

Predicted centromere regions were determined using a synteny based approach using the H99 reference genome (GCA_000149245.3) where centromere regions have been experimentally identified (38,39,99). The 5 kb regions flanking H99 centromeres were used as BLASTn (v2.14.0+) queries against assembled genomes for centromere mapping (100,101).

### Full-length TE mapping in assembled genomes

To estimate full-length TE copy numbers from assembled genomes BLAT (v35) and BLASTn (v2.14.0+) were used, full-length TE sequences obtained from linear amplicon long-read sequencing of TE insertions into *FRR1* were provided as queries (49). Full-length copies were defined as hits with identity ≥ 90% and alignment length ≥ 95% of the query length. For BLAT the −minIdentity=90 specification was used, and pslReps (v469) filtered hits to find the best alignment with a minimum coverage of 95% (−minCover=0.95). BLASTn hits meeting full-length criteria were filtered in R (v4.3.3). In instances were BLAT and BLASTn reported different full-length copy number estimates, the higher estimate was used. Only BLASTn was used to detect copies of CNL1 because BLAT reported overlapping CNL1 copies leading to overestimation, this was not observed for other TEs.

### Identification of structural rearrangements and synteny analysis

Whole-genome alignment was performed using Minimap2 (v2.24). Dotplots to visualize primary alignments between assemblies were generated in R (v4.3.3) using pafr (v0.0.2). Synteny and Rearrangement Identifier (SyRI) (v1.6.3) was used to define syntenic regions and structural rearrangements between serial isolates using default parameters (50). Plotsr was used to visualize syntenic regions (102).

### Southern analysis

Genomic DNA was prepared using a CTAB DNA extraction method (described above) and digested with restriction enzymes BspEI, PvuII, or PspXI for CNIRT6, MLDT, and KDZ1, respectively (New England Biolabs) (92). Digests were run on 0.7% agarose gels at 4°C for approximately 16 h. Fragments from the gel were transferred to a charged nylon membrane (Roche). Target probes were prepared using chemiluminescent DIG-labeling (Roche), see Table S5 for primers used to amplify probes. Hybridization of the DIG probes (DIG Easy Hyb Granules, Roche) and detection by chemiluminescence using an anti-DIG antibody (Anti-Digoxigenin-AP Fab Fragments, Roche) were performed according to the recommended manufacturer’s protocols and imaged on film (Amersham Hyperfilm ECL).

### TE annotation and repeat masking

Transposon annotation was performed using Earl Grey (v4.1.0) with default parameters (68). Repeat masking of insertions, deletions, and duplications identified by SyRI were analyzed using Genetic Information Research Institute (GIRI) Repbase CENSOR (103).

### Gene annotation

Gene annotations were lifted over from the H99 reference genome (GCA_000149245.3) by Liftoff (v1.6.3) with the -polish flag (104).

### TE copy number estimation using short reads

TE copy number estimation using short reads was performed as previously described (32). Briefly, publicly available short reads (PRJNA37160) were mapped to the H99 reference genome (GCA_000149245.3) using Minimap2 (v2.24), mosdepth (v0.3.10) was used to calculate the median read depth in 500 bp non-overlapping windows, the median depth reported was used for normalization (105). Short reads were also mapped using Minimap2 to a multi-fasta containing all full-length TE sequences identified inserting into *FRR1*. Mosdepth was then used to produce the cumulative read depth distribution. For each TE, read depth at 0.95 coverage was normalized by the genome-wide median read depth to estimate full-length copy number.

### RNAi gene loss-of-function prediction

Genome feature annotation was “lifted over” from the H99 reference genome (FungiDB R68) to each isolate-specific genome assembly using the software tool Liftoff (v1.6.3) (104). The `-polish` option of Liftoff was employed to re-align exons in cases where the lift-over procedure resulted in start/stop codon loss or introduced an in-frame stop codon. Based on the polished lift-over annotation, the AGAT GTF/GFF Toolkit software (https://github.com/NBISweden/AGAT) was used to predict protein sequences for all annotated genes in each strain-specific assembly using the àgat_sp_extract_sequences.pl` script. Where multiple protein isoforms are annotated in the reference genome, predictions were generated for each isoform. For each RNAi-related gene of interest, the predicted amino acid sequences for each isolate were compared to the H99 reference genome. Candidate loss-of-function alleles were classified as those encoding proteins whose predicted length is < 85% of the length in H99.

## Supplementary Material

Supplement 1

Supplement 2

Supplement 3

Supplement 4

Supplement 5

Supplement 6

Supplement 7

## Data Availability

Raw nanopore sequencing reads and whole-genome assemblies generated from isolates used in this study are available on NCBI’s Sequence Read Archive (SRA) under BioProject PRJNA1222431, accession numbers are listed in Table S3. Full-length TE sequences identified inserting into *FRR1* were uploaded to GenBank, accession numbers are listed in Table S2.
