# Supplementary material for: Temperature and genetic background drive mobilization of diverse transposable elements in a critical human fungal pathogen
Source: bioRxiv. 2025 May 23:2025.05.19.654958. Preprint. [Version 1] doi: 10.1101/2025.05.19.654958 (PMC12139950; doi:10.1101/2025.05.19.654958)
Supplement: Supplement 7 [file NIHPP2025.05.19.654958v1-supplement-7.pdf]

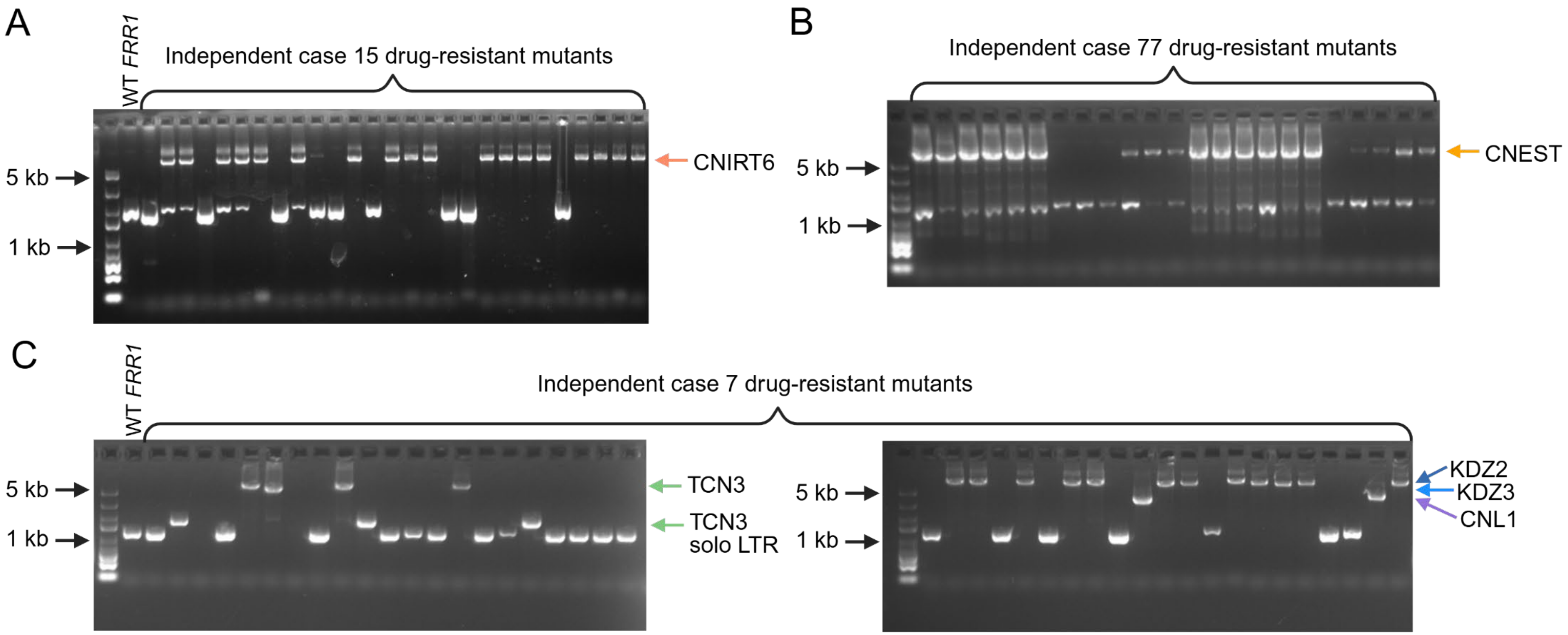

Figure S1. PCR amplification of *FRR1* in wild-type (WT) and independent rap+FK506-resistant mutants. *FRR1* was amplified from independent drug-resistant mutants from case 15 (**A**), case 77 (**B**), and case 7 (**C**); a subset of representative mutants is shown. Higher molecular weight bands indicative of TE insertion in *FRR1* were verified by Sanger or linear amplicon sequencing. Colored arrows indicate the molecular weights of bands identified as TEs.

bioRxiv preprint doi: https://doi.org/10.1101/2025.05.19.654958; this version posted May 23, 2025. The copyright holder for this preprint (which was not certified by peer review) is the author/funder, who has granted bioRxiv a license to display the preprint in perpetuity. It is made available under aCC-BY 4.0 International license.

A

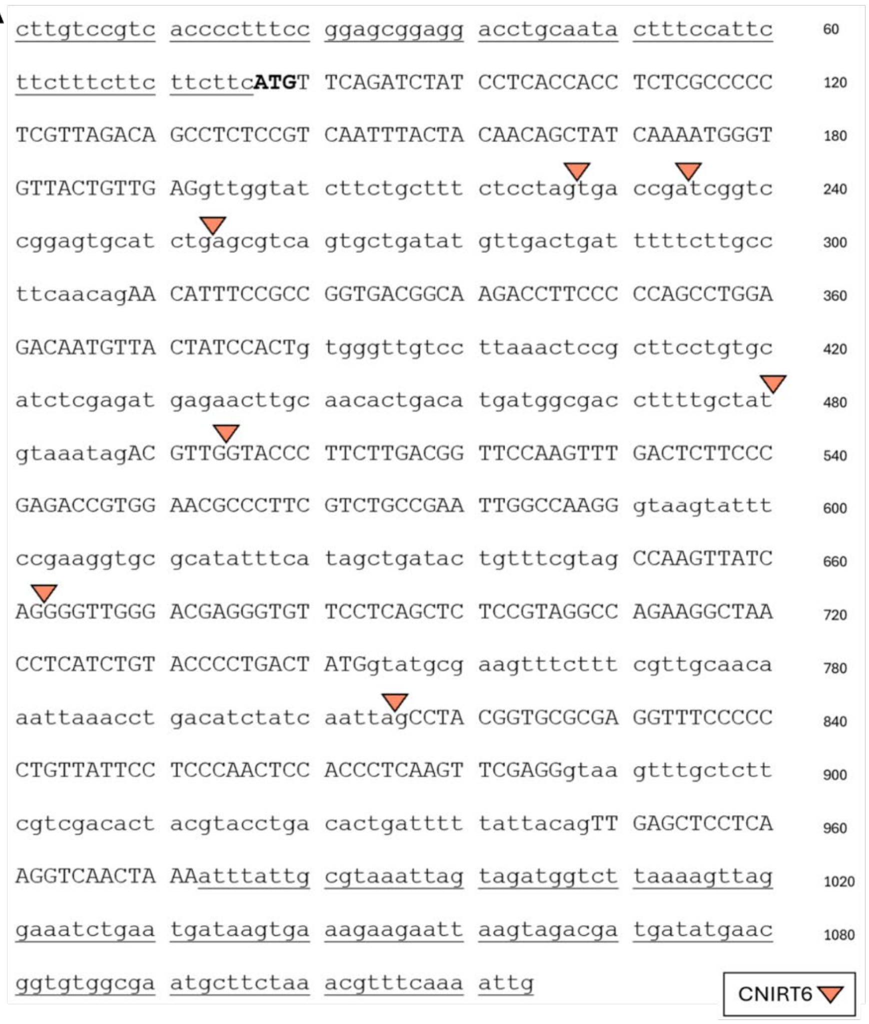

B

CNIRT6 Insertion	TSD	Size of TSD
1	TAG	3
2	CGA	3
3	CTG	3
4	TAT	3
5	TTG	3
6	CAG	3
7	TTA	3

Figure S2. CNIRT6 insertion into *FRR1*. (**A**) A subset of CNIRT6 insertion sites (orange arrows) mapped in *FRR1*. The 5′ and 3′ untranslated regions (UTRs) of *FRR1* are underlined; introns are shown in lowercase, exons in capital letters, and the start codon is bolded. (**B**) TSDs detected at CNIRT6 insertion sites. Insertions are ordered based on their appearance in the *FRR1* sequence from beginning to end.

bioRxiv preprint doi: https://doi.org/10.1101/2025.05.19.654958; this version posted May 23, 2025. The copyright holder for this preprint (which was not certified by peer review) is the author/funder, who has granted bioRxiv a license to display the preprint in perpetuity. It is made available under aCC-BY 4.0 International license.

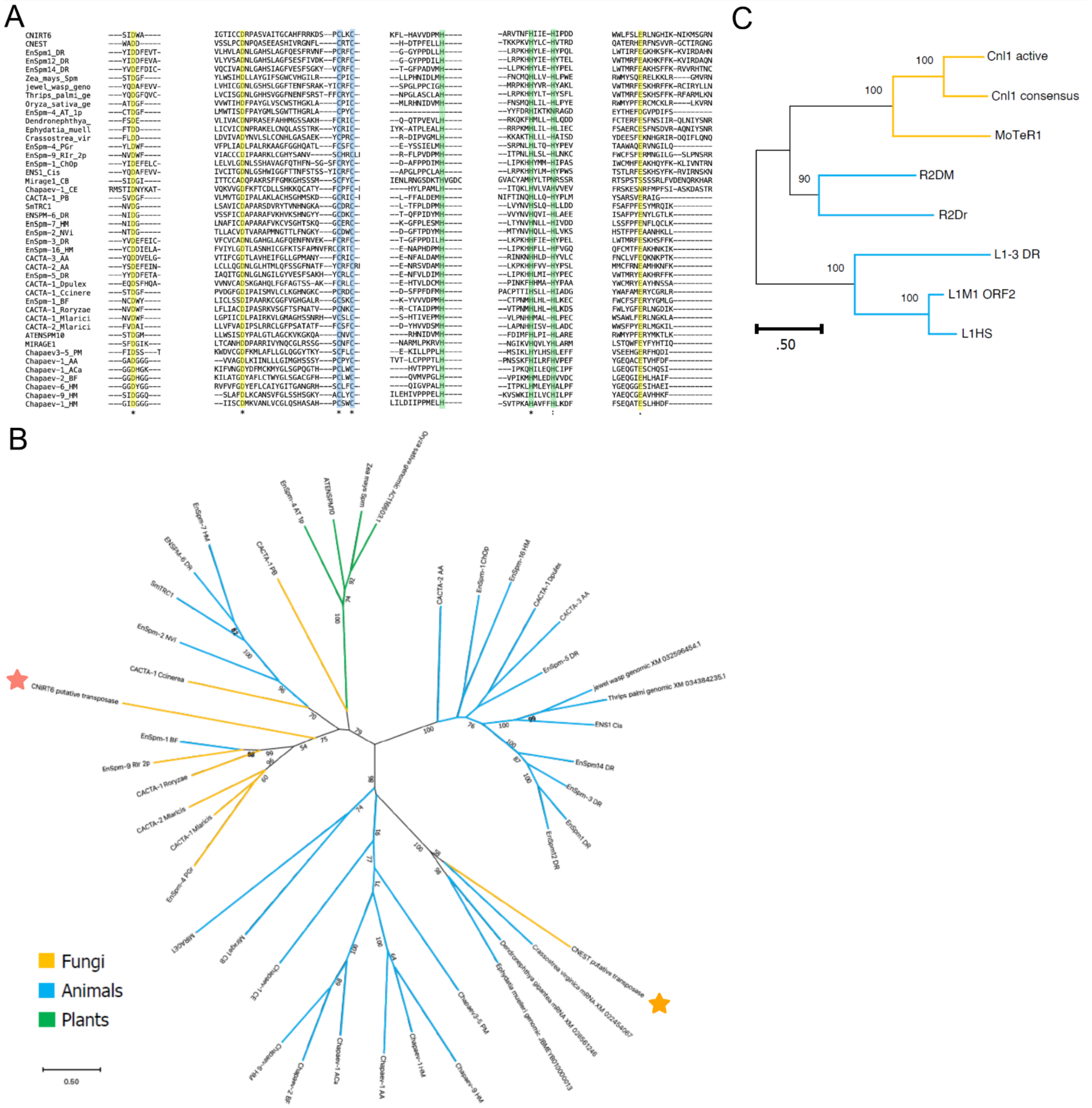

Figure S3. Classification of mobile CMC/EnSpm-like elements (CNIRT6, CNEST), and CNL1. (**A**) Regions of interest in a multiple sequence alignment highlighting conserved resides in CMC/EnSpm-like elements. Highlighted residues include the DDE catalytic core (yellow), conserved cysteine residues (blue) and conserved histidine residues (green) (42). (**B**) Unrooted amino acid phylogeny of diverse CMC/EnSpm transposases, demonstrating the relationship of CNIRT6 and CNEST with known CMC/EnSpm families and other related sequences. CNIRT6 is marked with an orange star, and CNEST with a gold star. (**C**) Unrooted amino acid phylogeny of reverse transcriptase domains, demonstrating the relationship between CNL1 and MoTeR1, and more distantly related R2 elements from flies and fish, as well as mammalian L1 elements. Branches are colored by kingdom: animals (blue), plants (green), and fungi (yellow). Bootstrap values greater than 50% are shown at nodes. Scale bars represent amino acid substitutions per site. Abbreviations: HS – *Homo sapiens*, DR – *Danio rerio*, DM – *Drosophila melanogaster*, CE – *Caenorhabditis elegans,* CB – *Caenorhabditis briggsae*, PM – *Petromyzon marinus*, BF – *Branchiostoma floridae*, , Dpulex – *Daphnia pulex*, Cis – *Ciona intestinalis*, Aca – *Aplysia californica*, Sm – *Schistosoma mansoni*, AA – *Aedes aegypt*, ChOp – *Chionoecetes opilio*, Jewel wasp – *Nasonia vitripennis*, Nvi – *Nasonia vitripennis*, HM – *Hydra magnipapillata*, Ccinerea – *Coproniopsis cinerea*, RIr – *Rhizophagus irregularis*, Roryzae – *Rizopus oryzae*, Mlaricis – *Melampsora laricis*, PGr – *Puccinia graminis*, PB – *Phycomyces blakesleeanus*, Mo – *Magnaporthe oryzae*, AT – *Arabidopsis thaliana*.

bioRxiv preprint doi: https://doi.org/10.1101/2025.05.19.654958; this version posted May 23, 2025. The copyright holder for this preprint (which was not certified by peer review) is the author/funder, who has granted bioRxiv a license to display the preprint in perpetuity. It is made available under aCC-BY 4.0 International license.

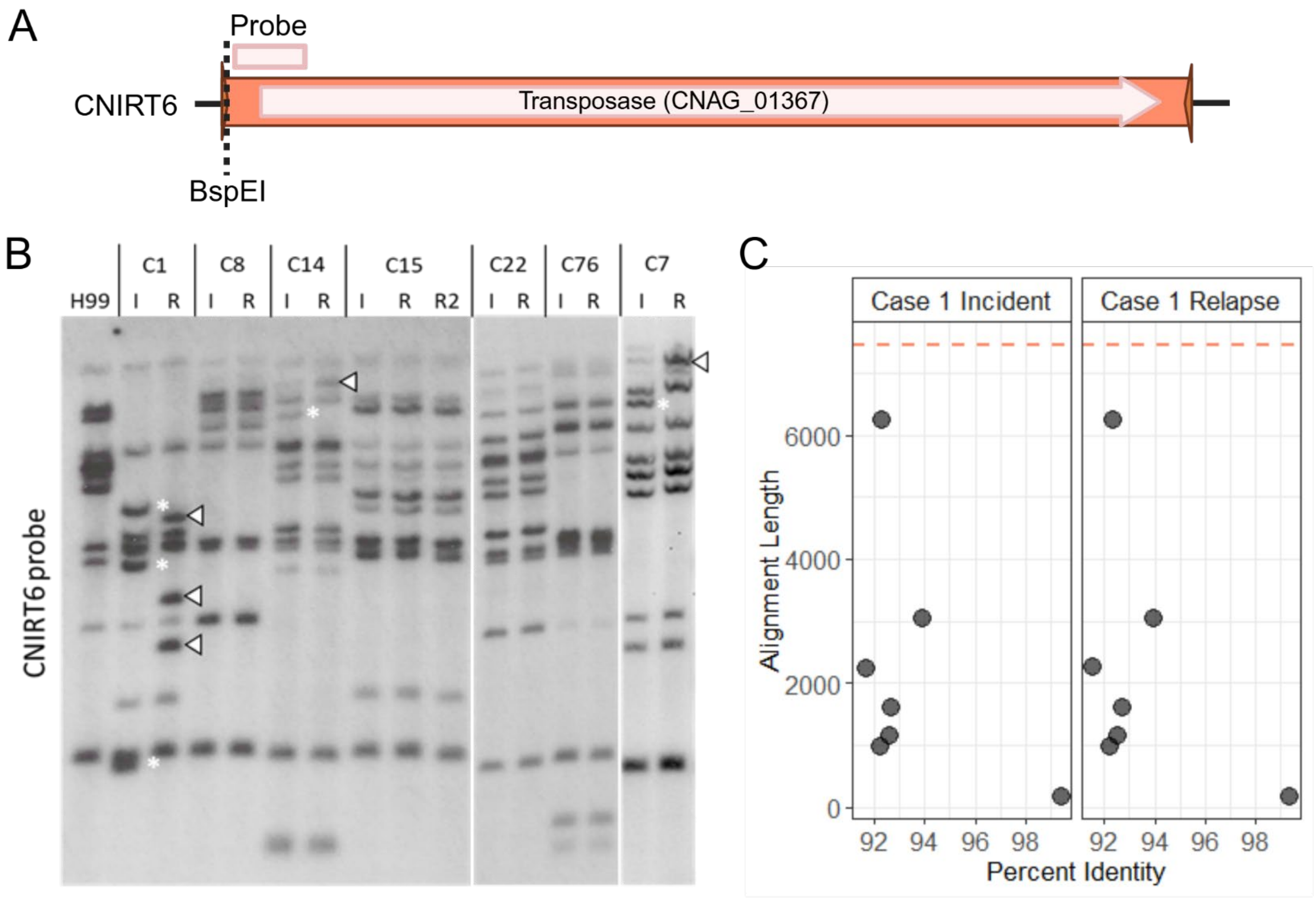

Figure S4. Putative CNIRT6 transposition events in serially collected clinical isolates. (**A**) Location of the restriction site (dotted line) and CNIRT6-specific probe used in Southern analysis. (**B**) Southern blot of genomic DNA digested with BspEI probed for CNIRT6 in incident (I) and relapse (R) isolates; case numbers are indicated as C#. Asterisks denote the loss of CNIRT6 fragments, while arrowheads indicate the emergence of CNIRT6 fragments at new genomic locations between incident and relapse isolates. (**C**) BLAT hits identified in case 1 isolates using full-length CNIRT6 as the query. The orange dashed line indicates the length of full-length CNIRT6.

bioRxiv preprint doi: https://doi.org/10.1101/2025.05.19.654958; this version posted May 23, 2025. The copyright holder for this preprint (which was not certified by peer review) is the author/funder, who has granted bioRxiv a license to display the preprint in perpetuity. It is made available under aCC-BY 4.0 International license.

A

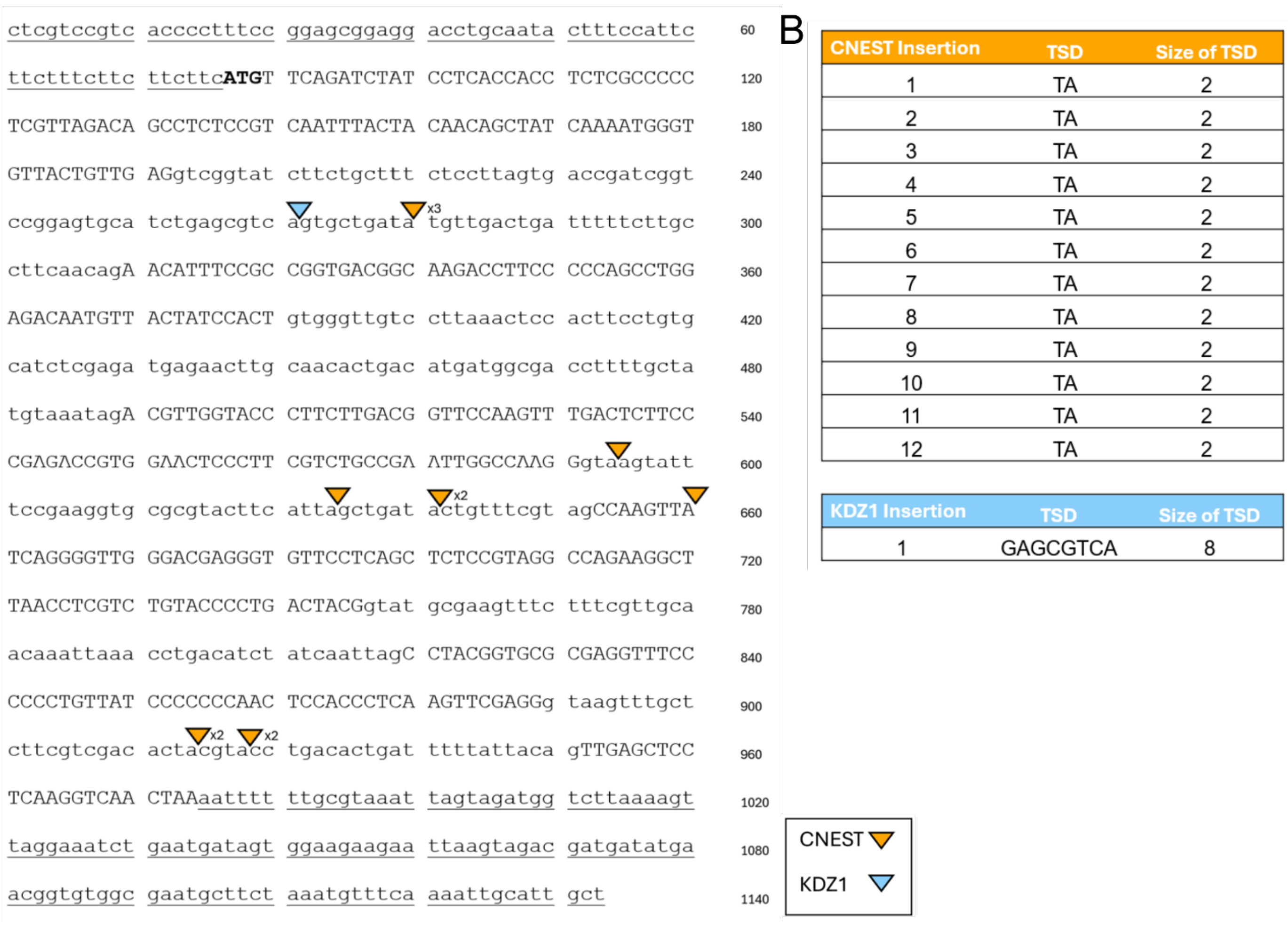

B

CNEST Insertion	TSD	Size of TSD
1	TA	2
2	TA	2
3	TA	2
4	TA	2
5	TA	2
6	TA	2
7	TA	2
8	TA	2
9	TA	2
10	TA	2
11	TA	2
12	TA	2

KDZ1 Insertion	TSD	Size of TSD
1	GAGCGTCA	8

Figure S5. CNEST and KDZ1 insertion into *FRR1*. (**A**) A subset of CNEST (gold) and KDZ1 (light blue) insertion sites are mapped with arrows in *FRR1*. The 5′ and 3′ untranslated regions (UTRs) of *FRR1* are underlined; introns are shown in lowercase, exons in capital letters, and the start codon is bolded. (**B**) TSDs detected at CNEST and KDZ1 insertion sites. Insertions are ordered based on their appearance in the *FRR1* sequence from beginning to end.

A

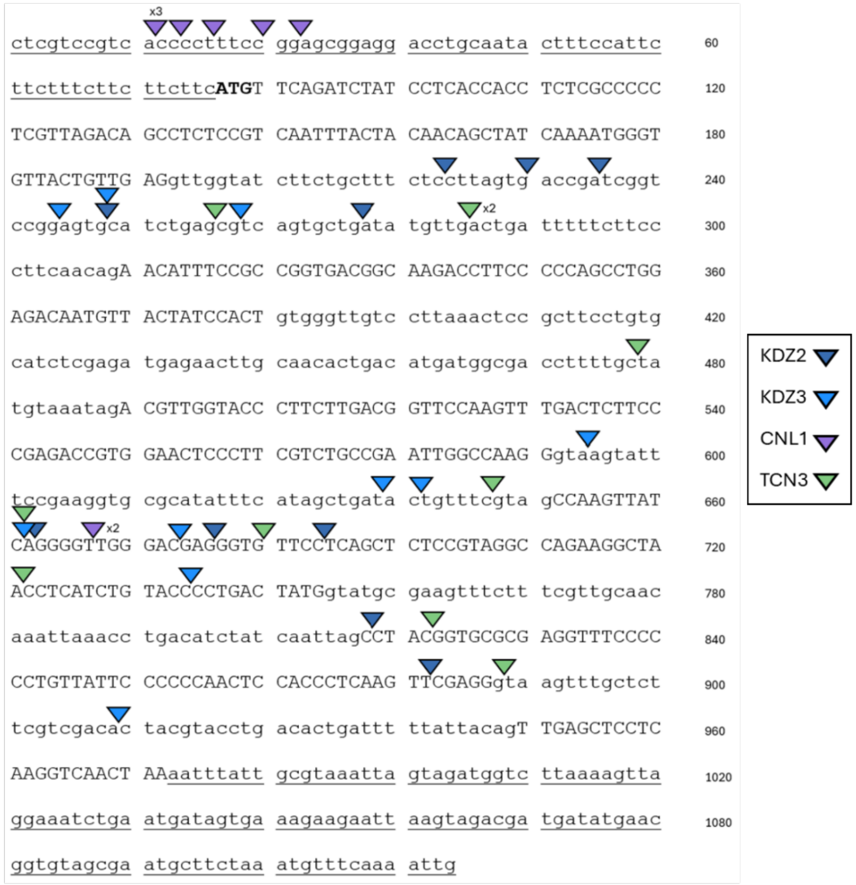

B

KDZ2 Insertion	TSD	Size of TSD
1	GCTTTCTC	8
2	CCTTAGTG	8
3	CCGA	4
4	CCGGAGTG	8
5	CAGTGCTG	8
6	AGTTATCA	8
7	GGGACGAG	8
8	GGTGTTCC	8
9	CAATTAGC	8
10	CTCAAGTT	8

KDZ3 Insertion	TSD	Size of TSD
1	CGGTCCGG	8
2	CCGGAGTG	8
3	TCTGAGCG	8
4	CAAGGGTA	8
5	TAGCTGAT	8
6	GCTGATAC	8
7	AAGTTATC	8
8	GTTGGGAC	8
9	TCTGTACC	8
10	GACA	4

CNL1 Insertion	TSD	Size of TSD
1	CA	2
2	A	1
3		0
4	CACC	4
5	CACCCCT	7
6	CACCCCTTTCC	11
7	CACCCCTTTCCGG	13
8		0
9	ATCAGGGGT	9

TCN3 Insertion	TSD	Size of TSD
1	CTGAG	5
2	TGTTG	5
3	TGTTG	5
4	TTTGC	5
5	GTTTC	5
6	TTATC	5
7	GGGTG	5
8	GCTAA	5
9	CCTAC	5
10	GAGGG	5

Figure S6. KDZ2, KDZ3, CNL1, and TCN3 insertion into *FRR1*. (**A**) A subset of KDZ2 (dark blue), KDZ3 (blue), CNL1 (purple), and TCN3 (green) insertion sites are mapped with arrows in *FRR1*. The 5′ and 3′ untranslated regions (UTRs) of *FRR1* are underlined; introns are shown in lowercase, exons in capital letters, and the start codon is bolded. (**B**) TSDs detected at KDZ2, KDZ3, CNL1, and TCN3 insertion sites. Insertions are ordered based on their appearance in the *FRR1* sequence from beginning to end.

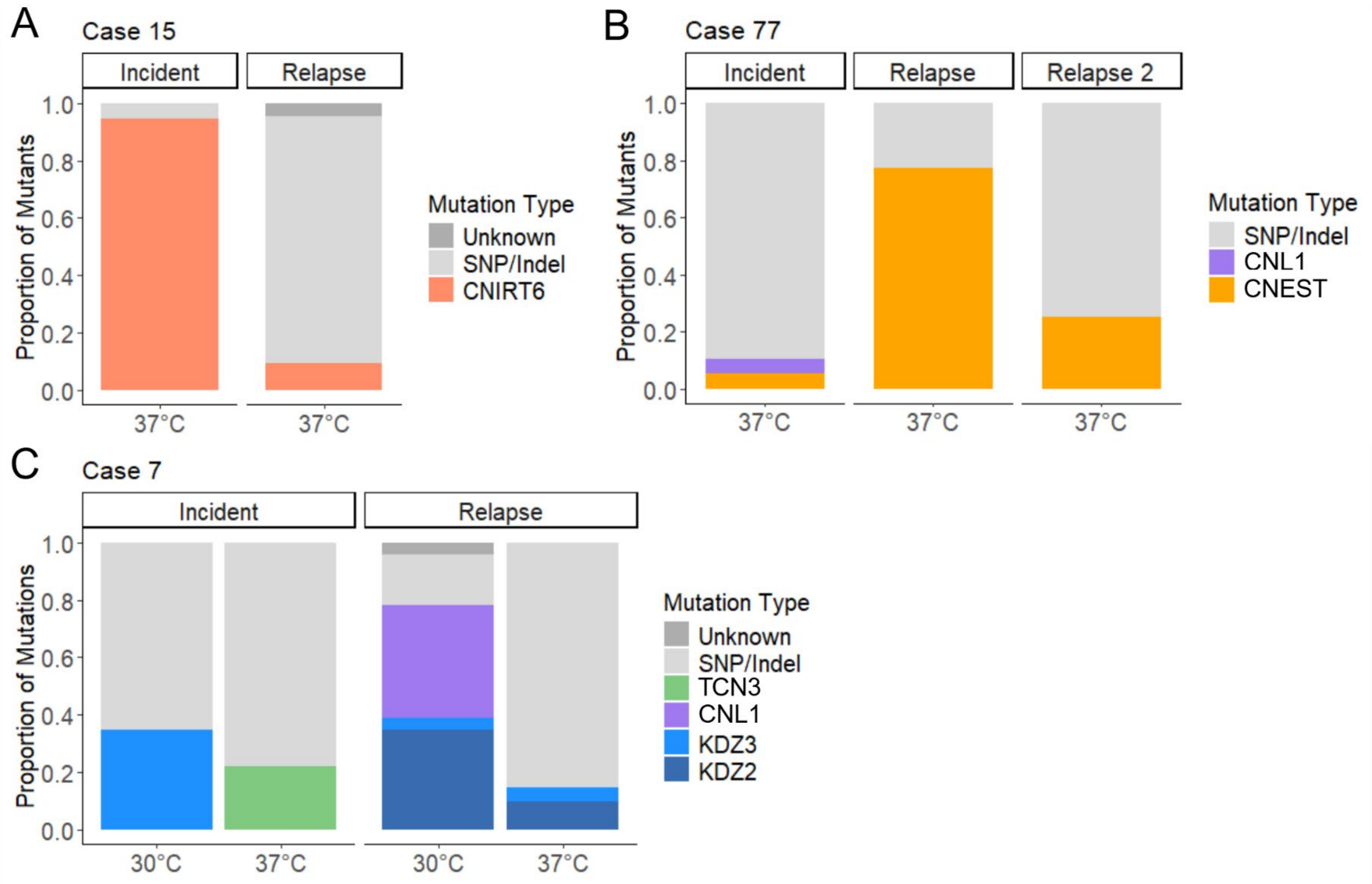

Figure S7. Limited nutrient availability alters TE mobilization dynamics. Mutation spectra in the *FRR1* gene for independent rap+FK506-resistant mutants in case 15 (**A**), case 77 (**B**), and case 7 (**C**). The mutation type "Unknown" indicates failure to amplify *FRR1* or absence of detectable mutations in its sequence (N = 18 – 31).

bioRxiv preprint doi: https://doi.org/10.1101/2025.05.19.654958; this version posted May 23, 2025. The copyright holder for this preprint (which was not certified by peer review) is the author/funder, who has granted bioRxiv a license to display the preprint in perpetuity. It is made available under aCC-BY 4.0 International license.

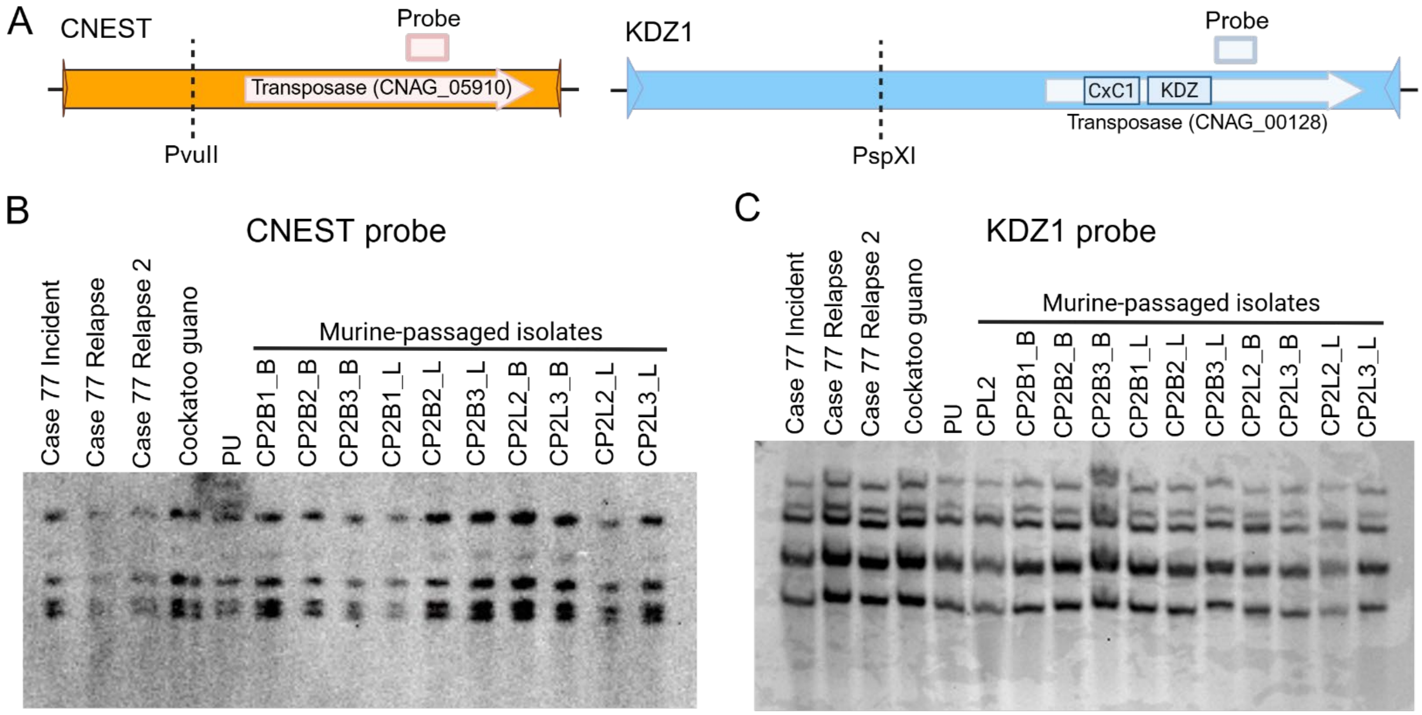

Figure S8. No predicted transposition of CNEST or KDZ1 in murine-passaged isolates. (**A**) Location of the restriction sites (dotted lines) and CNEST- and KDZ1-specific probes used in the Southern analysis. Southern blots of (**B**) PvuII-digested genomic DNA probed for CNEST, and (**C**) PspXI-digested genomic DNA probed for KDZ1. Shown are case 77 isolates, the cockatoo guano isolate, the patient isolate (PU) infected by the guano point-source strain, and cockatoo guano isolates passaged one to two times through mice by Sephton-Clark et al. (2023), recovered from brain or lung tissue (see Table S1 for strain details).

bioRxiv preprint doi: https://doi.org/10.1101/2025.05.19.654958; this version posted May 23, 2025. The copyright holder for this preprint (which was not certified by peer review) is the author/funder, who has granted bioRxiv a license to display the preprint in perpetuity. It is made available under aCC-BY 4.0 International license.

A

	CNIRT6	CNEST	KDZ1	KDZ2	KDZ3	CNL1	TCN3
Case 1 Incident	0	1	0	0	0	0	1
Case 1 Relapse	0	1	0	0	0	0	1
Case 7 Incident	0	1	1	3	2	16	2
Case 7 Relapse	0	1	1	3	2	39	2
Case 15 Incident	2	1	0	0	0	0	1
Case 15 Relapse	2	1	0	0	0	0	1
Case 15 Relapse 2	2	1	0	0	0	0	1
Case 77 Relapse	0	4	5	0	2	0	0
Case 77 Relapse 2	0	4	5	0	2	0	0
Cockatoo guano	0	4	5	0	1	0	0
H99	2	2	1	0	0	0	1

B

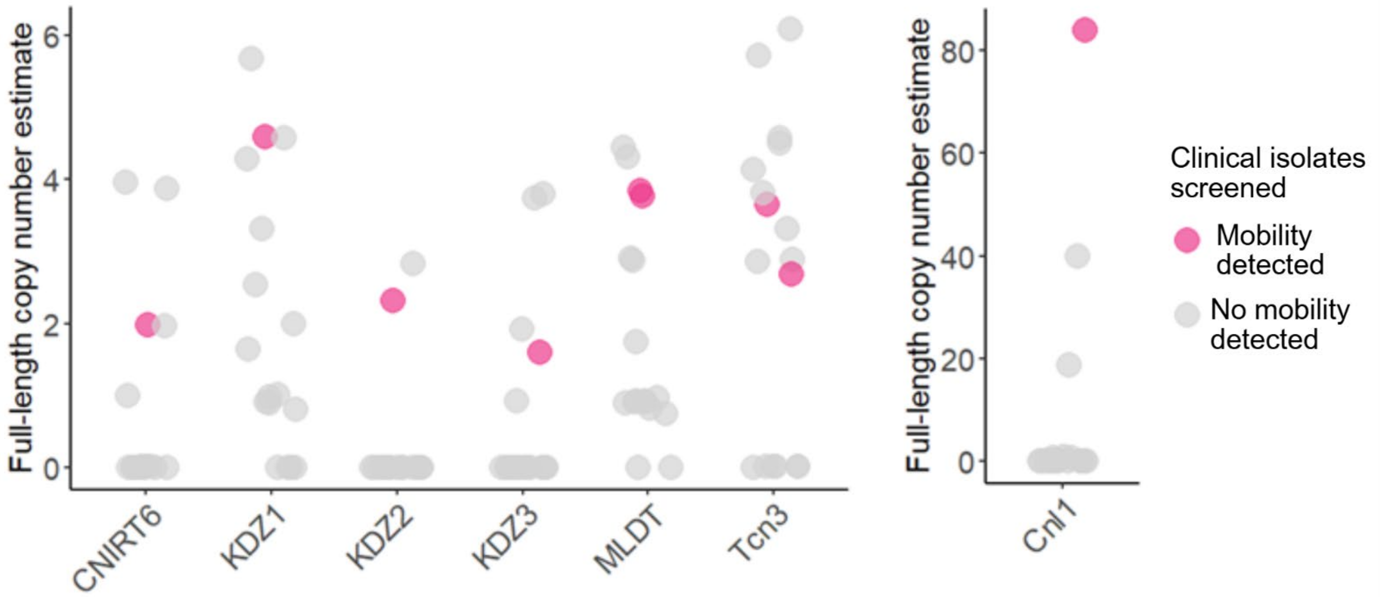

Figure S9. Full-length TE copies are present in genomes where no mobilization was detected. (**A**) Full-length TE copy numbers identified in whole-genome assemblies using BLAT and BLASTn homology searches. TE copy numbers in isolates with observed mobilization events are highlighted in pink. Isolate names are colored by lineage: VNI (blue), VNII (green), and VNBII (orange). (**B**) Full-length TE copy number estimates from short-read sequencing for all 15 *C. neoformans* incident isolates screened, isolates where no insertions were identified in *FRR1* are in grey, isolates where insertions were detected are in pink.

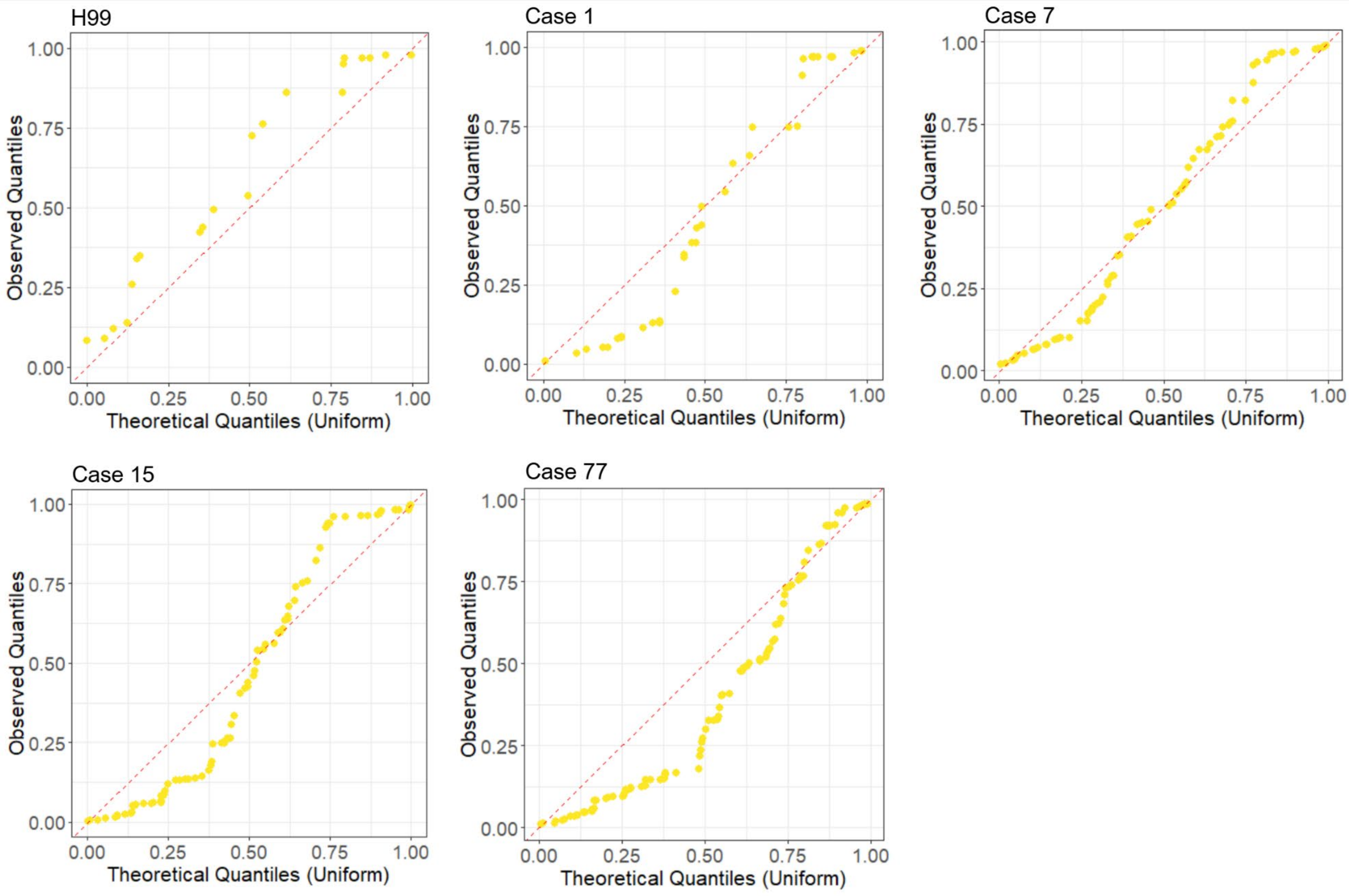

Figure S10. DNA TEs are approximately uniformly distributed across the genome. Quantile–quantile (QQ) plots are shown for the H99 reference genome and the first genome assembly from serially collected clinical isolates for cases 1, 7, 15, and 77 (representative examples). Observed quantiles correspond to the scaled positions (0 to 1) of DNA TEs along all chromosome arms. Theoretical quantiles are uniformly distributed random values between 0 and 1, sorted in ascending order, with their total number matching the number of observed DNA TEs.

bioRxiv preprint doi: https://doi.org/10.1101/2025.05.19.654958; this version posted May 23, 2025. The copyright holder for this preprint (which was not certified by peer review) is the author/funder, who has granted bioRxiv a license to display the preprint in perpetuity. It is made available under aCC-BY 4.0 International license.

A

Transposon type	Isolate	Total annotations	Percent methylated
LTR	Case 1	370	62.7
	Case 7	350	64.0
	Case 15	336	71.1
	Case 77	281	55.9
	**Median**	**343**	**63.4**
non-LTR	Case 1	25	8.0
	Case 7	223	53.4
	Case 15	74	8.1
	Case 77	204	37.7
	**Median**	**139**	**22.9**
DNA	Case 1	36	8.3
	Case 7	84	7.1
	Case 15	84	15.5
	Case 77	95	8.4
	**Median**	**84**	**8.4**

B

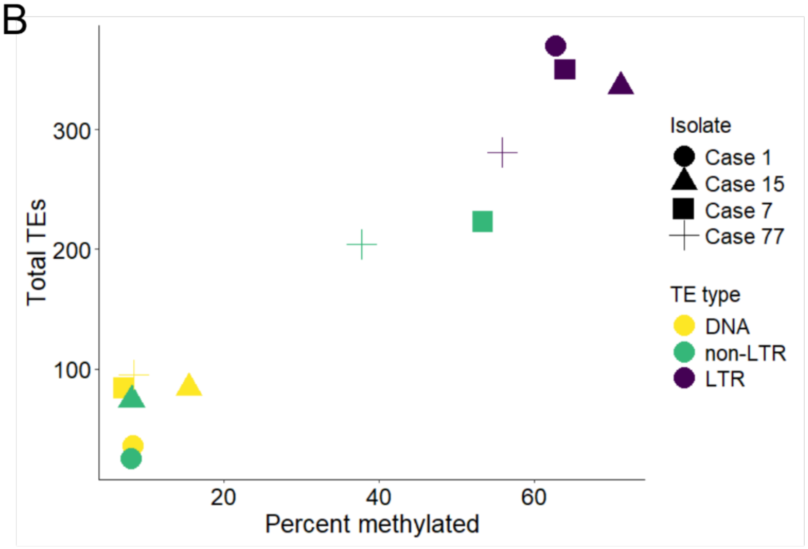

Figure S11. DNA transposons are less frequently methylated than LTR and non-LTR retrotransposons. (**A**) Number of TEs per type and the percentage containing at least one high-confidence DNA methylation call (methylation frequency > 0.75), with median values shown. (**B**) TE types (colors) per genome (shapes) and the percentage with at least one high-confidence DNA methylation call (methylation frequency > 0.75). The first genome assembly from serially collected clinical isolates was chosen as a representative.
